# Biochemometric 2D NMR-Based Heterocovariance Analysis: A Targeted Approach for Identifying Bioactive Compounds in Complex Mixtures

**DOI:** 10.1021/acs.analchem.5c02419

**Published:** 2025-10-12

**Authors:** Sigrid Adelsberger, Alexander F. Perhal, Lorenza Bertaina, Patrik F. Schwarz, Verena M. Dirsch, Judith M. Rollinger, Ulrike Grienke

**Affiliations:** † Division of Pharmacognosy, Department of Pharmaceutical Sciences, Faculty of Life Sciences, 27258 University of Vienna, Josef-Holaubek-Platz 2, 1090 Vienna, Austria; ‡ Vienna Doctoral School of Pharmaceutical, Nutritional and Sport Sciences, University of Vienna, Josef-Holaubek-Platz 2, 1090 Vienna, Austria

## Abstract

Biochemometric approaches, which integrate bioactivity data with spectroscopic or spectrometric data, offer significant potential to streamline the discovery of bioactive compounds in targeted isolation strategies. However, the complexity of natural extracts and the presence of structurally similar analogs make this process time-consuming and resource intensive. This study introduces a 2D nuclear magnetic resonance (NMR)-based heterocovariance analysis (HetCA) workflow to identify chemical features that correlate positively or negatively with bioactivity in complex mixtures. As a proof-of-concept, the workflow was established using artificially mixed samples of pentacyclic triterpenes which were screened for modulatory activities of the retinoic acid receptor-related orphan receptor gamma (RORγ) and the G protein-coupled bile acid receptor (TGR5). The validated concept was then exemplified using a triterpene-rich *Eriobotrya japonica* leaf extract. The applied workflow enabled the targeted and accurate identification of bioactive constituents from *E. japonica* that modulate RORγ and/or TGR5 using this newly developed biochemometric 2D NMR HetCA approach.

## Graphical abstract

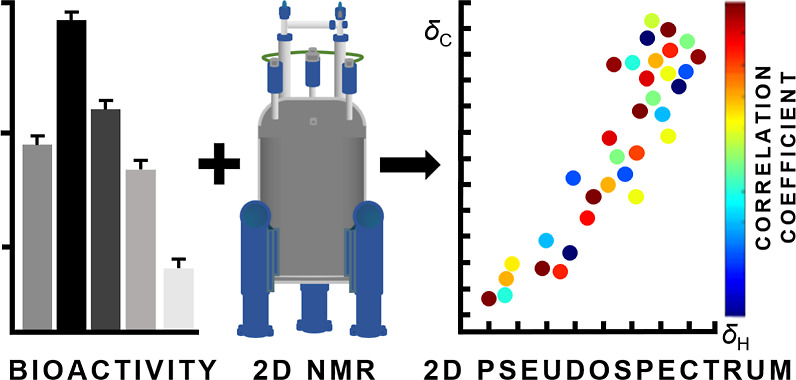

## Introduction

In search of new drug leads, natural products (NPs) are an important source^1^ with the disadvantage of having to deal with complex multicomponent crude extracts as starting materials.^2^ Biochemometric approaches allow the precise and targeted detection of bioactive constituents.^3–7^ The previously developed workflow ELINA (Eliciting Nature’s Activities)^8^ primarily relies on ^1^H NMR-based statistical heterocovariance analysis (HetCA),^9^ which detects specific chemical features, i.e. resonances, that are positively or negatively correlated with bioactivity prior to isolation.^10–13^

The interpretation of ^1^H NMR HetCA pseudospectra derived from mixtures of structural analogs with overlapping resonances pushes biochemometric methods to their limits.^14–16^ In particular, triterpenes (TTs) represent a class of compounds that includes many structural analogs.^17^ In addition to overlapping resonances, especially in the aliphatic region, TTs pose several analytical challenges, e.g. reduced sensitivity for UV absorption,^18^ low volatility,^19^ and poor ionizability in MS-based experiments.^20^ Hence, 2D NMR experiments are crucial for unambiguous structure determination of TTs.^8^

The retinoic acid receptor-related orphan receptor gamma isoform RORγt and the Takeda G protein-coupled receptor 5 (TGR5) were selected as suitable targets since their endogenous ligands show a structural similarity to the investigated compound class of tetracyclic and pentacyclic TTs.^21,22^ RORγ promotes the differentiation of naïve T cells into interleukin (IL)-17 producing T helper cells (Th17), which release pro-inflammatory cytokines such as IL-17 and IL-22.^21^ Small molecules can act as inverse agonists of RORγ thereby reducing cytokine production and inflammation.^23^ TGR5, also known as GPBAR-1 or M-BAR,^22^ is involved in the regulation of metabolic diseases and inflammation.^24^ When stimulated by agonists, TGR5 exerts anti-inflammatory effects via inhibition of transcriptional nuclear factor-κB (NF-κB) as well as modulating other signaling pathways.^25–27^

2D NMR techniques such as heteronuclear single quantum coherence (HSQC) have found their way into dereplication workflows.^28^ However, statistical correlation of 2D NMR with bioactivity data, while promising, is still in its infancy. The aim of this study was to move beyond dereplication and to unravel RORγ and TGR5 modulating TTs in mixtures in a targeted, and unambiguous manner. Therefore, a biochemometric 2D NMR-based HetCA approach was developed. As a proof-of-concept, artificially mixed TTs underwent biochemometric analyses and as an authentic application sample, a dichloromethane extract of TT-rich *Eriobotrya japonica* leaves was selected.

## Experimental Section

### Solvents and Reagents

All solvents (analytical grade), ammonium formate (HiPerSolv CHROMANORM, ≥99%), and ethanol (>99.7%) were purchased from VWR Chemicals (Radnor, USA). Chloroform-D1 (MagniSolv, 99.8%) was purchased from Merck (Darmstadt, Germany), carbon dioxide (CO_2_) 4.5 from Messer (Vienna, Austria), sulfuric acid (95.0–98.0%), vanillin (ReagentPlus, 99%), and Dulbecco's modified Eagle's medium (DMEM) (D6546) from Sigma-Aldrich (St. Luois, USA). Fetal bovine serum (FBS) (S1810) was purchased from Biowest (Nuaille, France), glutamine (BE17-605E), penicillin and streptomycin (DE17-602E) from Lonza Group AG (Basel, Switzerland), 5× reporter lysis buffer (E397A) from Promega (Fitchburg, USA), trypsin/EDTA from Thermo Fisher Scientific (Waltham, USA). Double-distilled deionized water (dd H_2_O) was produced by a Milli-Q-Plus ultrapure water device (Millipore Corporation, Bedford, MA, USA) and by an Arium Pro Ultrapure Lab Water System from Sartorius (Göttingen, Germany).

### Plant Material

Dried leaves of *E. japonica* (batch number 030724) were provided by Plantasia GmbH (Oberndorf/Salzburg, Austria). A voucher specimen (JR-20250218-A1) is stored at the Division of Pharmacognosy, Department of Pharmaceutical Sciences, University of Vienna, Austria.

### Extraction of *E. japonica* Leaves

An *E. japonica* dichloromethane extract (EJD) was prepared from 512.31 g dried, milled leaves with 4.5 L dichloromethane at room temperature on an orbital shaker GFL-3005 (GFL Gesellschaft für Labortechnik, Burgwedel, Germany). The plant material was split into two parts and put into 3000 mL Erlenmeyer flasks with 2250 mL dichloromethane each and was extracted four times (4 d, 2 d, 1 d, and 5 d). After filtration and solvent evaporation (Rotavapor R II, Büchi, Flawil, Switzerland) at 40 °C, the residue was dried in a desiccator, yielding 16.46 g of EJD.

### Fractionation and Isolation

EJD was fractionated by flash chromatography on an Interchim puriFlash 4250 instrument (Interchim, Montluçon, France) controlled by Interchim Software into 45 microfractions (MFs), named MF1–MF45. The device was equipped with a photo diode array (PDA) detector, an evaporative light scattering detector (ELSD), and a Silica HP 220 Gramm 25 μm 220 bar puriFlash column (PF-25SIHC-F0220, Interchim, Montluçon, France). 13.75 g of EJD were prepared for dry load application by mixing it with silica gel 60 (0.040–0.063 mm, CAS-No 7631-86-9, 60.08 g/mol, Merck KGaA, Darmstadt, Germany) in a ratio of 1:1. The gradient for *n*-hexane (A) and acetone (B), with a flow rate of 50.0 mL/min, was: 2% B for 3 column volumes (cv), 2% B to 5% B in 7 cv, 5% B for 5 cv, 5% B to 10% B in 7 cv, 10% B for 5 cv, 10% B to 15% B in 7 cv, 15% B for 5 cv, 15% B to 20% B in 7 cv, 20% B to 40% B in 5 cv, 40% B for 7 cv, 40% B to 100% B in 10 cv, 100% B for 7 cv. To combine in total 2121 test tubes with 20 mL each into 45 MFs, results of ultrahigh performance supercritical fluid chromatography (UHPSFC) and thin layer chromatography (TLC) were used. The fractionation of MF33 with the same device and detectors via a C_18_ HQ 12 Gramm 15 μm 22 bar column (PF-15C18HQ-F0012) was conducted with a flow rate of 15.0 mL/min with dd H_2_O (A) and acetonitrile (B): 5% B for 3 min, 5% B to 45% B in 4 min, 45% B to 98% B in 80 min, 98% B for 17 min, 98% to 5% B in 0 min, 5% B for 15 min. 124.20 mg of MF33 were homogenized 1:1 with 125.0 mg Silica gel 60 for dry load application. UHPSFC and TLC analyses were used to guide the pooling of 359 test tubes into eight fractions A1–A8. Fractionation of 30 mg A3 in methanol (25.29 mg/mL) was performed on a SFC Prep-15 device (Waters, Milford, MA, USA) equipped with an ELSD and PDA detector. A Torus 1-aminoanthracene column (OBD, 130 Å, 10 × 250 mm, 5 μm) (Waters, Milford, MA, USA) was kept at 45 °C. The mobile phase consisted of supercritical CO_2_ and ethanol absolute >99.7% with a gradient [time (min)/% B]: 0.0/10, 1.5/10, 16.5/11, 18.5/50, 20.5/50, 21.5/10, 22.5/10. Three fractions, named B1–B3, were collected according to the ELSD chromatogram. TLC was performed using silica gel 60 F_254_ plates (Merck, Darmstadt, Germany). The mobile phase consisted of methanol, dichloromethane and ethyl acetate with 10:1.2:0.5 v/v. The analysis was performed at visible light after derivatization with vanillin (1% in methanol) and sulfuric acid (5% in methanol) and temperature increase to 105 °C for 2 min.

### Ultrahigh-Performance Supercritical Fluid Chromatography (UHPSFC)

UHPSFC analysis of MF1–MF45 was performed on an Acquity UPC^2^ device (Waters, Milford, MA, USA) equipped with a PDA detector and ELSD. A Torus 1-aminoanthracene column (130 Å, 3.0 × 100 mm, 1.7 μm) (Waters, Milford, MA, USA) was kept at 45 °C. Supercritical CO_2_ (A) and methanol (B), flow rate 1.0 mL/min, were used for the gradient [time (min)/% B]: 0/10, 6.5/19, 7.5/50, 8.5/50, 9/10, 10/10. Samples were prepared with 5 mg/mL in *n*-hexane and 2-propanol 7:3. The measurements for A1–A8 and B1–B3 (5 mg/mL in acetonitrile and methanol) were performed in the same way with some changes. The gradient [time (min)/% B] was: 0/10, 15/13, 15.2/50, 16.2/50, 16.50/10, 17/10. Additionally, a single quadrupole mass spectrometry detector (QDa by Waters (Milford, MA, USA)) was used with a flow rate of 0.6 mL/min of 10 μM ammonium formate (in dd H_2_O/methanol 1:9) as makeup solvent. Both positive (15 V cone voltage) and negative mode (30 V) were recorded with a scan range from 100.00 to 1000.00 Da.

### Bioassays

#### Cell Culture

HEK293 cells (CRL-1573, ATCC) were grown in DMEM supplemented with 10% FBS, 2 mM glutamine, 100 U/mL penicillin, and 100 μg/mL streptomycin. Cells were cultured at 37 °C and 5% CO_2_ and passaged every 2–3 days, with a maximum use up to passage number 35.

#### RORγ-Gal4 Luciferase Reporter Gene Assay

A hybrid RORγ-Gal4 construct was employed, in which the RORγ ligand-binding domain (LBD) is fused to the Gal4 DNA-binding domain (DBD), a yeast transcription factor that drives luciferase expression through a yeast upstream activating sequence, as previously described.^29^ Briefly, 8 × 10^6^ HEK293 cells were transfected with the calcium phosphate method using 5 μg of Gal4-receptor (RORγ-Gal4: Dr. Fabio R. Santori, Center for Molecular Medicine, University of Georgia, Athens, Georgia, USA), 5 μg of response element (tk­(MH1000)­4xLuc: Prof. Ronald Evans, Salk Institute for Biological Studies, La Jolla, California, USA), and 3 μg of eGFP plasmid (pEGFP-N1: Clontech, Mountain View, CA, USA), and incubated overnight. Medium was changed, and cells were further incubated for 5 h before resuspension in 5% charcoal-stripped FBS-DMEM. Cells were then reseeded into a 96-well plate (5 × 10^4^ cells/well) and treated with compound solutions prepared in the same medium for 18 h. After cell lysis in 5× reporter lysis buffer, luminescence and fluorescence values were measured on a TECAN Spark Microplate Reader (TECAN Group AG, Männedorf, Switzerland). Luminescence signals were normalized to fluorescence emissions and expressed as fold activations normalized to the vehicle control dimethyl sulfoxide (DMSO) 0.1%.

#### CRE-Luciferase Reporter Gene Assay

As an indirect measurement of TGR5 receptor activation by compound treatment, CRE-luciferase reporter gene assays were performed as previously described.^30^ Briefly, HEK293 cells stably expressing an EPAC-based FRET biosensor as well as the TGR5 receptor (TGR5 HEK EPAC) were seeded at a concentration of 8 × 10^6^ cells on 150 mm cell culture dishes 4–5 h prior to transfection. Cells were then transfected with 10 μg CRE-luciferase reporter plasmid overnight. On the next day, cells were allowed to recover from transfection for 4–5 h in fresh medium before detachment from dishes using trypsin/EDTA. Cell suspensions were diluted in stripped (5% charcoal-stripped FBS) DMEM to a concentration of 5 × 10^4^ cells per well onto a 96-well plate and treated with vehicle control (0.1% DMSO), positive control LCA (10 μM) or compounds at the indicated concentrations for 18 h. Following treatment, the cells were lysed in 5× reporter lysis buffer. The basal fluorescence emission of the stably transfected EPAC FRET biosensor was used for cell number normalization and measured at an emission wavelength of 520 nm (excitation wavelength at 485 nm) using a TECAN Spark Microplate Reader (TECAN Group AG, Männedorf, Switzerland). Luminescence values (RLU) were measured and normalized to the respective fluorescence values (RFU) to account for differences in cell numbers (RLU/RFU) and normalized to the vehicle control (0.1% DMSO). Results are expressed as fold activations relative to vehicle control.

#### Statistics

Data obtained in the RORγ-Gal4 luciferase reporter gene assays and the CRE-luciferase reporter gene assays were checked for normal distribution via D'Agostino & Pearson calculations. For nonparametric data (as was the case for MF13 in Figure S13 only), Mann-Whitney-*U*-test was performed. Statistical analysis included one-way ANOVA and Dunnett's posthoc test (****p ≤ 0.0001, ***p ≤ 0.001, **p ≤ 0.01, *p ≤ 0.05, n. s. *p* > 0.05) versus vehicle control DMSO 0.1%. Data analysis was performed with Microsoft Excel version 2404 (Microsoft Corporation, Redmond, USA) and GraphPad Prism version 10.2.3 (403) (Dotmatics, Boston, USA). Results are expressed as mean ± standard deviation measured in *n* biological replicates. Nonlinear regression was used for IC_50_ value calculation.

### NMR Sample Preparation

Compounds **1**, **7**, **8**, **12**, **13**, AMF1–AMF8, MF33–MF35, and B1–B3, were dissolved in deuterated chloroform-*d* with 3 mg/mL. 750 μL of each sample were centrifuged at 3000 rpm for 5 min. 650 μL of the supernatant were transferred to an NMR tube (LabScape, Bruker, Germany).

### NMR Data Acquisition

All NMR experiments were acquired on a spectrometer consisting of a Bruker Ascend 500 MHz magnet (Bruker BioSpin, Billerica, MA, USA), a 5 mm triple-resonance Prodigy CryoProbe, a SampleJet automated sample changer, and an AVANCE NEO console. All measurements were performed at 298 K. The receiver gain was kept constant to ensure that the obtained spectra can be compared quantitatively. The resonance frequency was 500.19 MHz for ^1^H NMR and 125.77 MHz for ^13^C NMR. ^1^H NMR spectra were acquired using a standard Bruker pulse sequence program with default settings (zg30) and 128 scans. The acquisition was performed with FID resolution = 0.183 Hz, pulse width (PW) = 3.3 μs (corresponding to 1/3 of the 90° pulse length of 9.9 μs), relaxation delay (D1) = 1.5 s (in addition to the acquisition time of 5.45 s), size of real spectrum (SI) = 64k, and spectral width (SW) = 6009.615 Hz. The total acquisition time for each ^1^H NMR experiment was ∼15 min. ^1^H–^13^C HSQC NMR spectra were recorded using a standard Bruker pulse sequence program with default settings (hsqcetgpsisp2) and the following parameters: 4 scans, D1 = 2 s, a SW of 25.2 kHz and an acquired spectral size of 256 data points for ^13^C, and 7.5 kHz and 2048 data points for ^1^H. The total acquisition time for each experiment was ∼37 min. For all samples, both spectra were recorded. For structure elucidation of B1– B3, in addition to ^1^H NMR spectra and ^1^H–^13^C HSQC, ^13^C APT, ^1^H–^13^C HMBC, and ^1^H–^1^H COSY experiments were also conducted (parameters see Table S8).

### NMR Data Processing

Using TopSpin (Version 4.1.4, Bruker BioSpin GmbH, Rheinstetten, Germany, 2022), the NMR spectra were referenced to the residual nondeuterated solvent signals (δ_Η_ 7.26; δ_C_ 77.16), and underwent the protocol as described before.^8,9^ In brief, all FIDs were Fourier transformed, phased manually, followed by fifth order polynomial baseline corrections according to the mathematical equation *A* + *Bx* + *Cx* ^2^ + *Dx* ^3^ + *Ex* ^4^. Considered signal regions for all ^1^H spectra were defined as δ_H_ 0.5–10.0, for ^1^H–^13^C HSQC spectra of MF33–35 δ_H_ 0.0–8.0 and δ_C_ 0–150 and for AMF1–AMF4 δ_H_ 0.0–10.0 and δ_C_ 0–150.

### Heterocovariance Analysis (HetCA)

Spectral features from ^1^H NMR and ^1^H–^13^C HSQC data sets were statistically correlated with the bioactivity data on RORγ and TGR5 via HetCA in MATLAB (R2020a Update 4, 9.8.0.1417392, The MathWorks GmbH., Munich, Germany).

#### 1D HetCA

Statistical correlations were performed according to established protocols,^8,9^ using a bucket width of 0.0005 ppm. The HetCA plots visualize the determined covariance coefficient (cc), calculated via normalization of the covariance, with positively correlated signals pointing upward and negatively correlated signals pointing downward. The gradation of the cc between −1.0 and 1.0 for all HetCA experiments was depicted by MATLAB default color-code type jet from dark blue to dark red. For 1D HetCA, the results are shown with δ_H_ resonances on the *x*-axis and the cc on the *y*-axis.

#### 2D HetCA

^1^H–^13^C HSQC data sets were statistically correlated with the bioactivity data on RORγ and TGR5 in MATLAB for the calculation of 2D HetCA pseudospectra. As a first step of the 2D HetCA workflow, peak picking for the HSQC spectrum of the first microfraction of a selected HetCA package was performed in TopSpin. Within the data directory of the respective HSQC data, the generated peaklist.xml file was retrieved. This file was then copied into the respective HSQC data folder of the second microfraction of the package. Then, again peak picking was performed for the second microfraction. New peaks were appended, whereas duplicates of peaks were discarded from the peaklist.xml file. This extended peak list was then copied into the respective folder of the HSQC data of the next microfraction within the selected package. These steps were repeated for all microfractions of a package. The so obtained peak list was then used to collect the intensities for the cross-peaks of each HSQC within the package. Therefore, the generated peaklist.xml file was copied into the data directory of each microfraction of a package. Individual intensities of all cross-peaks of all microfractions of a package were obtained by using the “reset intensities – complete table” option in TopSpin. Peak picking of ^1^H–^13^C HSQC cross-peaks for 2D HetCA of AMF1–AMF4 was conducted with a maximum of 100 picked peaks, and for MF33–35 with a maximum of 500 picked peaks, with at least 2% of the highest intensity. Some signals, that were not ideally captured during automatic peak picking, were picked manually. Afterward, cross-peak δ_H_ and δ_C_ values together with their corresponding intensities for all microfractions of a package were collected in one excel file and the data set was sorted by ascending δ_H_ values. This final feature list was imported into MATLAB. After integrating the respective bioactivity data, the HetCA process correlates them with the NMR resonance intensities, in accordance with established 1D HetCA protocols.^8,9^ Instead of a bucket list, the information of the final feature list was used for MATLAB HetCA calculations. The correlation between activity and the intensities of each cross-peak were calculated. The resulting cross-peak specific correlation coefficient (cc) values were presented as a HSQC pseudospectrum, with the δ_H_ and δ_C_ values in ppm on the *x*-axis and left *y*-axis, and the right *y*-axis representing the calculated cc. Each pseudo cross-peak was additionally color coded according to the respective cc, between −1.0 and 1.0 from dark blue to dark red, using MATLAB default jet colormap. In the 2D HetCA pseudospectra, the visual representation of the generated cross-peaks as dots was scaled to a uniform size.

## Results and Discussion

### Selection of Compounds and Preparation of Artificial TT Mixtures for a Proof-Of-Concept Study

A proof-of-concept study was set up to evaluate whether a 2D NMR-based biochemometric approach is superior to 1D techniques and suitable for the differentiation of active and inactive structural analogs with overlapping ^1^H NMR resonances. For this purpose, 15 known active as well as known inactive TTs were selected deliberately: ursolic acid (**1**), oleanolic acid (**2**), echinocystic acid (**3**), erythrodiol (**4**), uvaol (**5**), α-amyrin (**6**), faradiol (**7**), betulin (**8**), lupeol (**9**), maslinic acid (**10**), corosolic acid (**11**), hederagenin (**12**), bayogenin (**13**), asiatic acid (**14**), and ganoderic acid A (**15**) (Chart 1 and Table S1).

**Chart 1:**
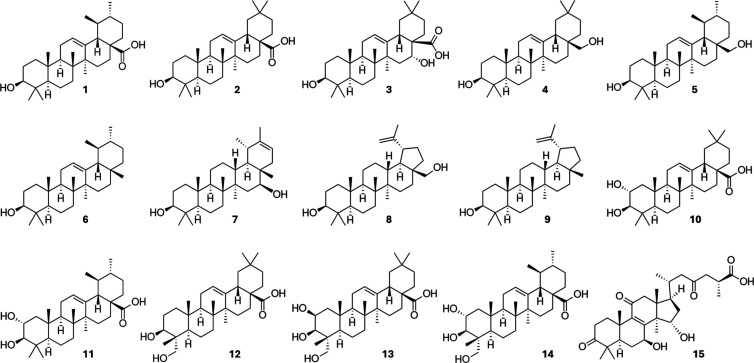
Chemical Structures of 15 Selected Triterpenes (TTs). Ursolic Acid (**1**), Oleanolic Acid (**2**), Echinocystic Acid (**3**), Erythrodiol (**4**), Uvaol (**5**), α-Amyrin (**6**), Faradiol (**7**), Betulin (**8**), Lupeol (**9**), Maslinic Acid (**10**), Corosolic Acid (**11**), Hederagenin (**12**), Bayogenin (**13**), Asiatic Acid (**14**), and Ganoderic Acid A (**15**)

To obtain consistent bioactivity data independent of already described activities, all 15 TTs were tested in the same assay systems. For evaluating the activity on RORγ, a Gal4 luciferase assay was employed, and for testing the activity on TGR5, a stable TGR5-expressing cell line with a CRE luciferase reporter gene were used. Based on these results, which were in accordance with the literature (Table S2) the 15 selected TTs were classified into three groups covering active, moderately active, and inactive TTs (Figure S1). To keep the proof-of-concept study simple, five out of the 15 TTs were selected to cover the three preassigned bioactivity groups on both targets: **1** (active), **12** (moderately active), and **7**, **8**, **13** (inactive) (Figures S2–S7) were used to prepare mixtures in different quantitative ratios to obtain eight artificial microfractions (AMF1–AMF8) simulating chromatographic fractionation (Table S3). All AMFs were subjected to bioactivity assessment on RORγ and TGR5 in luciferase reporter gene assays as well as 1D and 2D NMR measurement. Based on the bioactivity results, it was possible to define microfraction packages with increasing or decreasing bioactivity, e.g. AMF1–AMF4 for the target TGR5 showing a distinct sequentially decreasing bioactivity (Figure 1).

**Figure 1:**
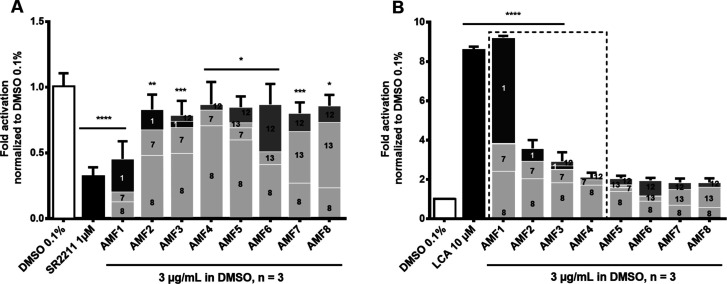
Bioactivities of artificial TT mixtures AMF1–AMF8 (3 μg/mL) on the targets (A) RORγ in RORγ-Gal4 luciferase assays and (B) TGR5 in CRE-luciferase assays. The color coding within the individual bars reflects the bioactivity of the respective compound (dark gray: active, medium gray: moderately active, light gray: inactive) of the mixed TTs, and each colored section is labeled with the respective compound number. The size of a section within a bar represents the relative amount of a compound in the corresponding AMF. Package AMF1–AMF4 was used for HetCA (marked with a dashed rectangle).

### 1D HetCA Pseudospectra

To perform HetCA, two prerequisites must be met: (i) a bioactive crude extract must be fractionated into microfractions (MFs) that contain an intersecting quantitative variance of constituents, and (ii), this quantitative variance must be reflected in a correlated ascending or descending variance of bioactivity.^8^ ^1^H NMR experiments of the selected microfraction package (AMF1–AMF4) were recorded as basis for the 1D HetCA of the proof-of-concept study. To ensure consistent signal-to-noise ratios, samples were prepared in the same way and measured under the same conditions (Figure S8). For the statistical correlation between chemical features and bioactivity data, the raw ^1^H NMR spectral data were processed in MATLAB according to a standard protocol,^8,9^ to obtain color-coded pseudospectra with positively (red, upward signals) or negatively (blue, downward signals) correlated resonances based on the correlation coefficient (cc), calculated via normalization of the covariance (Figure 2).

**Figure 2:**
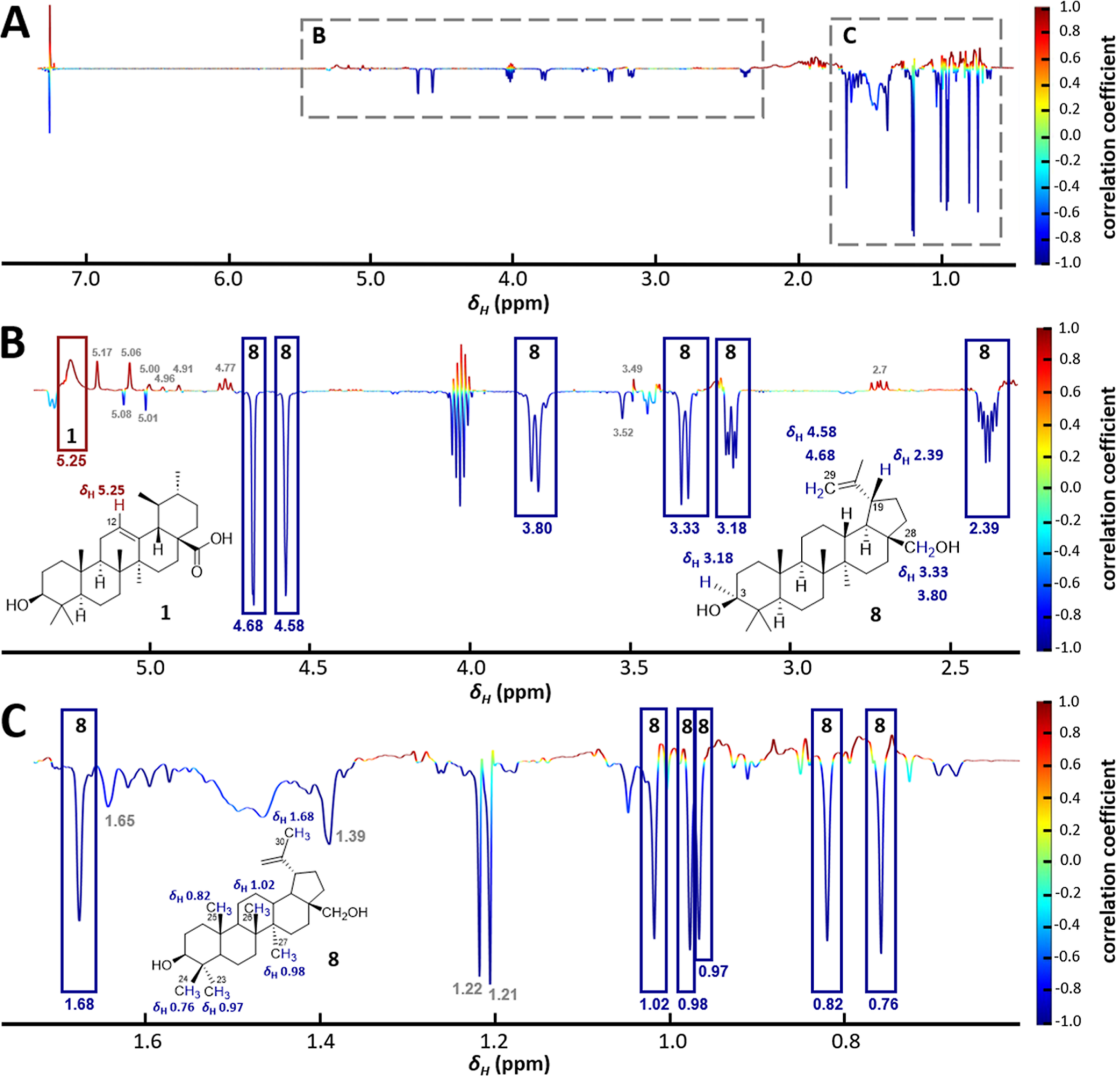
(A) 1D HetCA pseudospectrum of AMF1–AMF4 depicting TGR5 bioactivity with δ_H_ resonances on the *x*-axis and the correlation coefficient on the *y*-axis. (B) Zoom into the region δ_H_ 2.30–5.50 and assignment to the involved TTs **1** and **8**. (C) In the aliphatic region, only negatively correlated signals related to **8** could be determined.

The compounds contained in the MFs possess no aromatic structures, only a few alkenyl hydrogens, and many aliphatic protons. Thus, resonances in the chemical shift region δ_H_ 2.5–6.0 are limited, while the upfield region (δ_H_ 0.0–2.5) is rather crowded with overlapping signals. Based on the 1D HetCA results, **1** was statistically determined to be active, and **8** to be inactive. This is consistent with the experimental TGR5 bioactivity data. **7** and **12** could not be assigned to any noticeable signals in the 1D pseudospectrum and were therefore not covered by 1D HetCA. Quantitatively, **8** was the main component in the AMF1–AMF4 set, based on the high intensity of its proton resonances. Due to overlapping signals, the 1D HetCA analysis provided no further insight.

### 2D HetCA Pseudospectra for the Proof-Of-Concept Study

To overcome the limitations of the 1D NMR-based approach, a 2D HetCA plot for the same sample set (AMF1–AMF4) was generated based on ^1^H–^13^C HSQC measurements (Figures S9–S12). To calculate HetCA with 2D NMR spectra, peak picking was performed for each individual spectrum. All cross-peak intensities together with their δ_H_ and δ_C_ values were collected in one file and imported into MATLAB. The next step was to import the bioactivity values for each microfraction package. HetCA was then performed in the same way as for the 1D spectra.^8,9^ Finally, the 2D HetCA pseudospectrum was visualized, again using a color-code to show the cc. Independent of the original intensity, each cross-peak in the 2D HetCA spectrum was normalized to the same size so that all calculated signals have the same visibility. This means that each calculated pseudo cross-peak is represented graphically as a data point of uniform size, minimizing the risk of overlooking less intense signals.

Lessons learned from the 1D HetCA results were considered when preparing the data for the 2D analysis. For instance, essential marker signals in the 1D pseudospectrum, such as δ_H_ 5.25 for **1** and δ_H_ 3.18 for **8**, were specifically targeted for 2D HetCA. The 2D HetCA of AMF1–AMF4 resulted in a 2D pseudospectrum with δ_H_ values on the *x*-axis, δ_C_ values on the left *y*-axis and the color-coded cc on the right *y*-axis (Figure 3).

**Figure 3:**
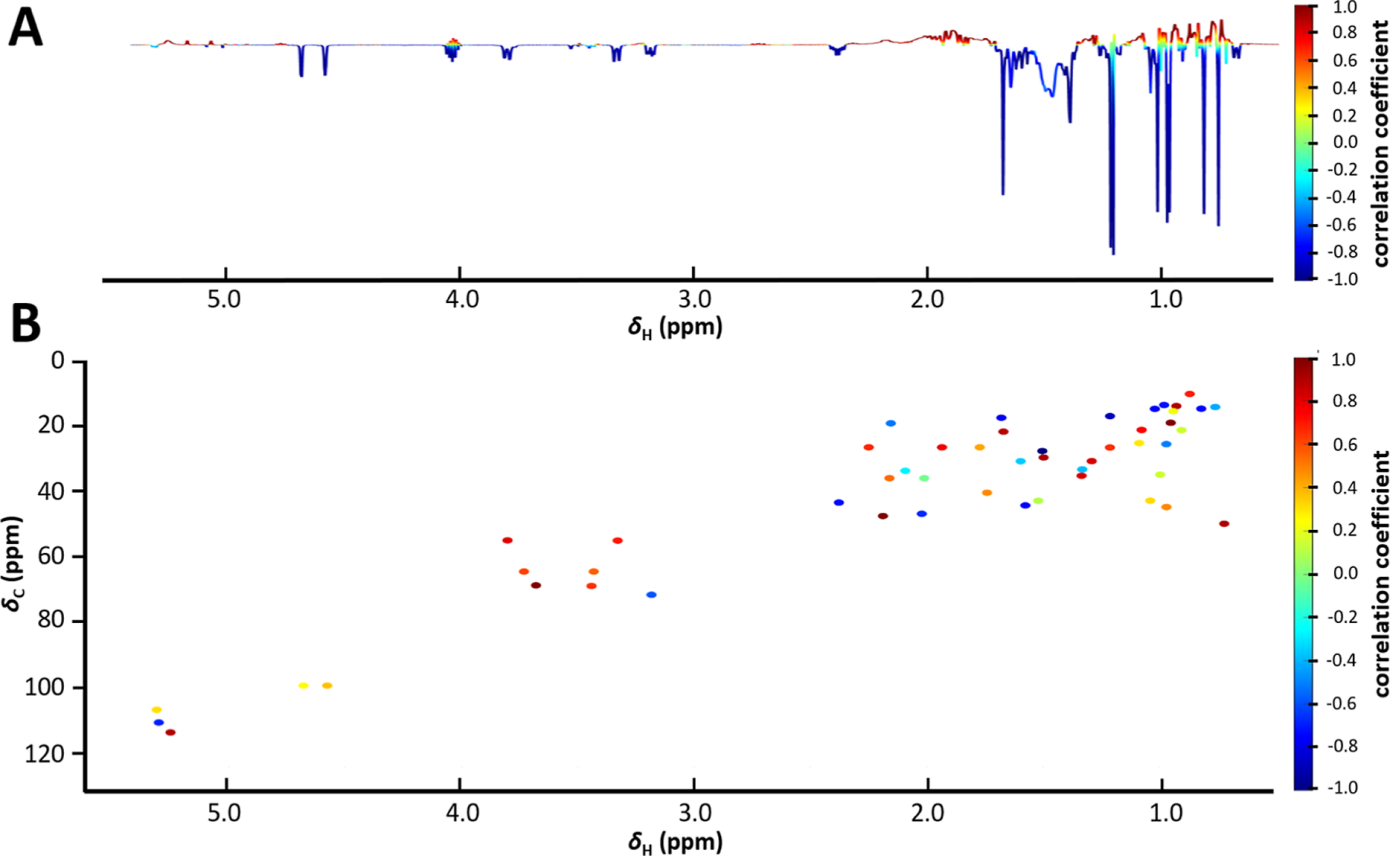
^1^H-based 1D HetCA pseudospectrum (A) and ^1^H–^13^C HSQC-based 2D HetCA pseudospectrum (B) for TGR5 bioactivity of AMF1–AMF4. The *x*-axis and left *y*-axis of the 2D plot show the δ_H_ and δ_C_ values in ppm, the right *y*-axis represents the calculated correlation coefficient, color-coded from active (dark red), to inactive (dark blue) signals. Listed δ_H_, δ_C_ value pairs see Table S4.

2D HetCA plots display signals based on δ_H_, δ_C_ values, enabling a clearer interpretation due to a distinction between similar δ_H_ values that overlap in the 1D HetCA plot. In the δ_H_ 0.0–2.5 region, it allowed better differentiation of active and inactive signals. **1** could be assigned as positively correlated based on red cross-peaks at δ_H_, δ_C_ 0.72, 55.0; 0.93, 15.2; 0.95, 20.9; 1.08, 23.3; 1.33, 38.8; 1.50, 32.7; 1.67, 23.9; and 2.19, 52.5. **8** was confirmed inactive by blue cross-peaks at δ_H_, δ_C_ 0.76, 15.1; 0.82, 16.1; 0.97, 28.1; 0.98, 14.9; 1.02, 16.3; 1.60, 48.9; and 2.38, 47.9, but two false calculations occurred (1.21, 29.3 (dark orange) and 1.93, 29.2 (medium red)). The moderately active status of **12** was demonstrated by signals of different colors at δ_H_, δ_C_ 0.87, 11.1 (light red); 0.90, 23.4 (green); 0.94, 16.9 (green); 0.97, 49.5 (orange); 1.29, 33.9 (medium red), 1.74, 44.6 (orange), 1.77, 29.2 (orange), 2.01, 39.7 (green), and 2.16, 39.7 (orange). **7** could be correctly assigned as inactive based on signals at δ_H_, δ_C_ 1.04, 47.3 (yellow); 1.33, 36.7 (light blue); and 2.10, 37.2 (turquoise).

In the δ_H_ 3.00–5.50 region of the 1D HetCA plot, the signals at δ_H_ 4.58, 4.68, 3.80, and 3.33, belonging to **8**, were correctly calculated as negatively correlated. In the 2D plot, the cross-peaks calculated for these resonances are displayed as signals with a weak or moderate positive correlation with activity (yellow, orange). Additionally, two 2D HetCA calculations in the same range were incorrect. The δ_H_, δ_C_ value pair 5.30, 122.0 belonging to the moderately active compound **12** was predicted to be inactive (blue), and the δ_H_, δ_C_ value pair 3.44, 76.1 belonging to the inactive compound **7** was calculated to be moderately active (orange). In the same ppm area, the 2D approach correctly identified **12** as moderately active (based on δ_H_, δ_C_ 3.73, 71.2; 3.68, 75.9; and 3.43, 71.2), and **1** as active (based on δ_H_, δ_C_ 5.25, 126.0).

Of 51 signals in the 2D plot, 34 were correctly classified as active, moderately active, or inactive, five were ambiguous, and 12 incorrect. Dark red cross-peaks appeared only for active compound **1** and moderately active compound **12**, while nine of 14 dark blue cross-peaks matched inactive compound **8**. 1D HetCA only enabled the detection and correct TGR5 bioactivity classification of **1** and **8**, 2D HetCA provided the mapping and classification of **1**, **7**, **8**, and **12**. Not only was the dispersion and visibility of the signals in the 2D HetCA improved, but also a greater variation of the cc color code gradations was observed. Notably, a subsequent targeted isolation would focus on the correct NMR signals of the 2D HetCA.

### Evaluation of 1D and 2D HetCA Using a Botanical Extract

To test the applicability of 2D HetCA for complex extracts, in the second part of this study, both 1D and 2D HetCA were applied and compared for the analysis of bioactive TTs in *E. japonica*.^31^ The plant contains at least 47 pentacyclic TTs^31,32^ many of which differ only in one or a few functional groups,^31^ making the search for bioactives using conventional methods challenging.^3^ Therefore, the aim was to determine whether 2D HetCA allows to fish out compounds with RORγ bioactivity in a targeted way, and to compare 2D results with those of the 1D plot. Dried leaves of *E. japonica* were macerated with dichloromethane at room temperature to obtain an extract (EJD). EJD was fractionated by normal-phase flash chromatography into 45 microfractions (MFs), designated MF1–MF45, and tested for their inverse agonist activity on RORγ in luciferase reporter gene assays at a concentration of 3 μg/mL.

In total, 10 microfraction packages were generated for consecutive MFs based on their ascending or descending activities on RORγ (Figure S13). All packages underwent UHPSFC-MS analysis and preliminary compound annotation by comparison with literature data.^31,33,34^ The most promising packages with partially unknown constituents were prioritized for the 1D and 2D NMR HetCA approach. Although the microfraction package MF33–35 contains known bioactive TTs such as **1** and **2** (Figure S14), it was selected due to additional peaks that could not be assigned to reference compounds (Figure S15). Therefore, ^1^H NMR and HSQC spectra of MF33–35 were recorded (Figures S16–S19) and processed according to the protocol described for the proof-of-concept study.

### 1D and 2D NMR-Based HetCA Analyses of MF33–35

The 1D NMR-based HetCA analysis revealed positively correlated and negatively correlated ^1^H NMR resonances associated with activity on RORγ; the 2D NMR-based HetCA calculations yielded 207 cross-peaks with color coding ranging from dark red to dark blue (Figure 4).

**Figure 4:**
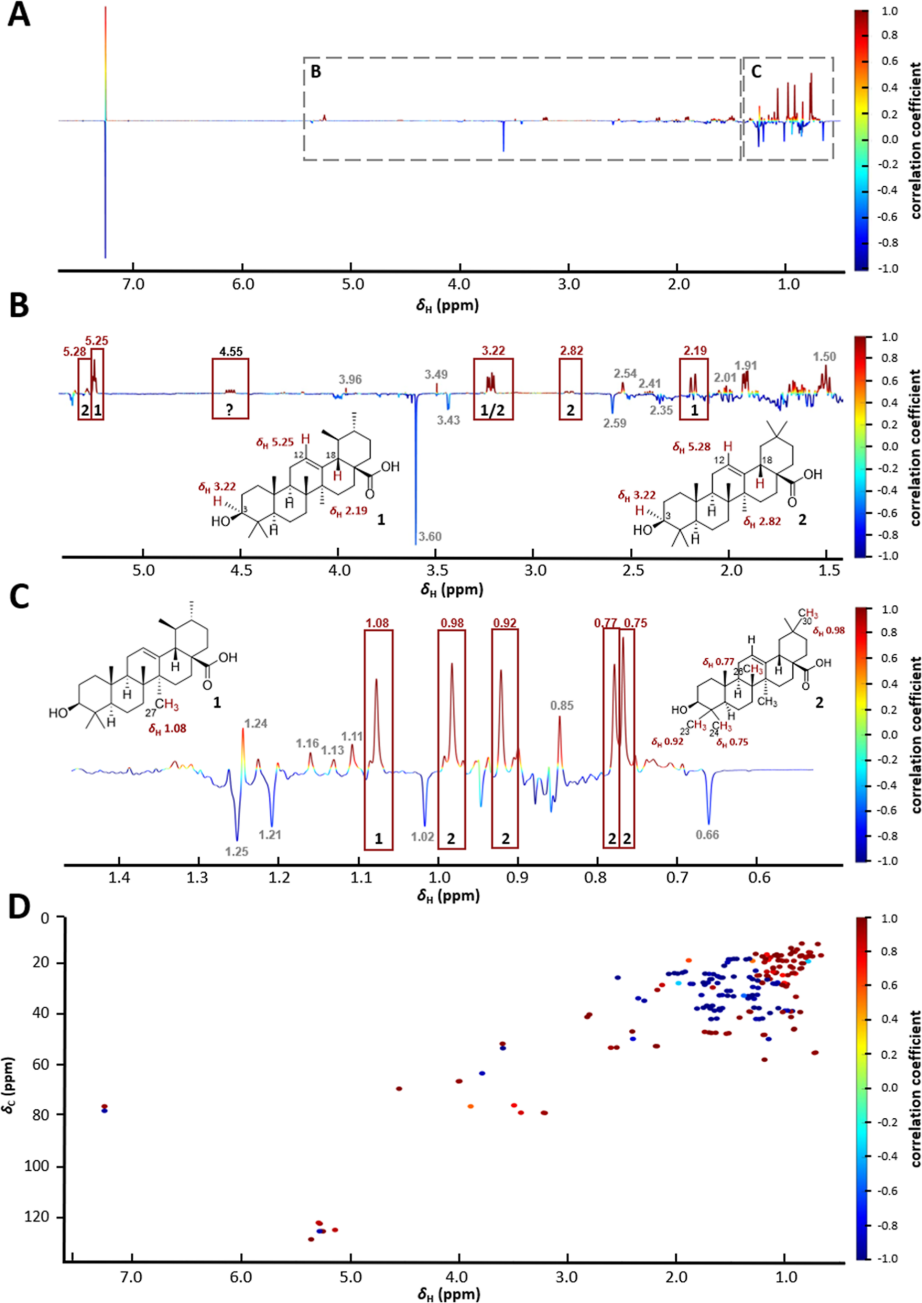
1D HetCA pseudospectrum of MF33–35 based on RORγ activity (A), with enlarged regions δ_H_ 1.50–5.05 (B) and δ_H_ 0.50–1.50 (C). (D) 2D HetCA pseudospectrum, based on ^1^H–^13^C HSQC data, with δ_H_ values on the *x*-axis, δ_C_ values on the left *y*-axis, correlation coefficient on the right *y*-axis. Positively correlated signals shown in dark red, negatively correlated signals in dark blue.

While many positively correlated features in the 1D plot matched the known bioactive compounds **1** and **2**, others remained unassigned. A positively correlated doublet of doublets at δ_H_ 4.55 showed no correspondence to any reference TT and was used as a marker feature in subsequent 2D NMR-based HetCA and targeted isolation. In the 2D NMR-based HetCA, various positively and negatively correlated δ_H_, δ_C_ pairs (Table S5) were observed, mostly at extreme cc values (1.0, dark red and −1.0, dark blue). Comparison with TT references **1** and **2** provided insights into the possible chemical structures responsible for the respective cross-peaks. Signals in the region between δ_H_, δ_C_ 5.14–5.36, 125.2–128.9 likely arise from alkene moieties, while those in the region δ_H_, δ_C_ 2.18–4.55, 41.0–78.9, could be assigned to protons of secondary alcohol carbons or tertiary protons at C-18 adjacent to a carboxyl group. Additional cross-peaks may originate from methylene groups adjacent to hydroxyl groups, next to the C-12 double bond or to a carboxyl group, as well as from tertiary protons (e.g., at C-9 or C-5), and various methylene and methyl groups.

To filter out the cross-peaks with the highest positive correlation to activity in the 2D pseudospectrum, signals with a cc > 0.990 were evaluated. Among the 14 δ_H_, δ_C_ pairs identified, the marker feature at δ_H_ 4.55 (δ_C_ 69.3), reappeared in this list. These signals spanned the entire 2D pseudospectrum (Table S6), suggesting diverse structural origins near different functional groups. None of the 14 features could be assigned to reference compounds.

All signals previously assigned in 1D HetCA also appeared as positively correlated with bioactivity in the 2D HetCA plot, with cc values between 0.934 and 0.985. The marker feature at δ_H_, δ_C_ 4.55, 69.3 again showed the highest value (0.9916). Several methyl proton cross-peaks had strong correlations (cc > 0.97), including δ_H_, δ_C_ 0.86, 21.9; 0.85, 21.7; 0.85, 19.7; 0.84, 19.7; 0.79, 17.0; 0.75, 17.0; and 0.70, 12.1. Unlike in 1D HetCA, the 2D plot allowed clearer differentiation of overlapping signals, mostly present in the aliphatic region, and a graded assessment of bioactivity. All 14 selected features, including the marker signal at δ_H_, δ_C_ 4.55, 69.3, showed higher signal intensity in MF33 compared to MF34 and MF35, leading to its selection for targeted isolation of the underlying putative RORγ-modulating compound.

### Phytochemical Processing of MF33

MF33 was fractionated by flash chromatography into fractions A1–A8, which were subjected to ^1^H NMR experiments to follow the marker signal at δ_H_ 4.55. At this stage, **1** and **2** could already be separated (Figure S20). The NMR marker feature was detected in fractions A3–A5 (Figure S21). Since A3 contained the highest amount of the target compound, it was further fractionated with semipreparative SFC into B1–B3 (Figure S22). Only in the ^1^H NMR spectrum of B1, the signal at δ_H_ 4.55 could be detected (Figure S23).

1D and 2D NMR experiments and comparison with the literature^34,35^ revealed the target compound to be 2α,19α-dihydroxy-3-oxo-12-ursen-28-oic acid (**16**) (C_30_H_46_O_5_, molecular weight 486.69 g/mol) with a purity of 95.8% based on ELSD. Methyl groups between δ_H_ 0.80–1.27 are clearly distinguishable, and the marker signal at δ_H_ 4.55 is assigned to C-2 (δ_C_ 69.3), which is bonded to a hydroxyl group (Table S7). This rare pentacyclic TT has already been described as constituent of *E. japonica* ^31,34^ with antitumor and anti-HIV effects.^31^ There have been no reports of its activity on RORγ. In addition, B2 and B3 were confirmed as 3-epimaslinic acid (**17**) and 3-epicorosolic acid (**18**) by comparison with previously reported spectral data,^36,37^ with a purity of 88.9% and 94.1% based on ELSD results.

### Bioactivity Testing of Compounds **16**, **17**, and **18** on RORγ

To validate the findings of the HetCA approach, compounds **16**, **17**, and **18** were tested in RORγ-Gal4 luciferase reporter gene assays. Additionally, a concentration–response curve and the IC_50_ of 2.15 μM was determined for compound **16** (Figure S24).

The results of the bioactivity studies confirmed the calculations of the 1D and 2D HetCA approach. The follow-up of the signal at δ_H_, δ_C_ 4.55, 69.3 led to the isolation of the bioactive compound **16**. Compounds **17** and **18** which do not share this marker feature showed less activity. Furthermore, the determined IC_50_ value of compound **16** in this study was lower than the RORγ activity previously reported by Zhou et al. for three main bioactive pentacyclic TTs known from *E. japonica*, namely methyl corosolate (6.51 μM), uvaol (**5**) (4.25 μM), and oleanolic acid (**2**) (8.59 μM).^33^ Of the 14 most active cross-peaks derived from the 2D HetCA pseudospectrum, six belonged to **16** (5.36, 128.9 at C-12; 4.55, 69.3 at C-2; 1.70, 47.1 at C-9; 1.24, 24.5 at C-27; 1.19, 57.8 at C-5; 1.03, 28.2 at C-15) and two signals belonged to **18** (5.27, 125.7 at C-12; 1.02, 28.4 at C-23). This highlights the potential of the 2D HetCA approach for the targeted identification of bioactives in complex mixtures based on biochemometric data mining.

## Conclusions

In this study, a 2D HetCA approach was developed using an artificially mixed set of TTs. In a follow-up application, 2D HetCA pinpointed to the bioactive compound **16** in a dichloromethane extract of *E. japonica* leaves (compound **16** content in EJD: 1.98%; Figure S25). This demonstrates the ability of the 2D HetCA approach to identify compounds that might otherwise be overlooked, even when they exhibit similar bioactivity to compounds present at higher concentrations. Although evaluating NMR data in 2D requires longer measurement times and more data interpretation, 2D HetCA provides a reduced spectral overlap due to the second dimension (δ_C_) and access to information that cannot be deduced from 1D HetCA pseudospectra. Further advantages include the finely subdivided gradation of the color coding and the size standardization of the cross-peaks in the pseudospectrum, which makes even less intense signals more visible.

In summary, 2D HetCA is a useful complement to the 1D HetCA approach and provides a valuable tool in the search for bioactive compounds in a targeted, resource-efficient and unambiguous manner, to reduce the number of necessary bioactivity tests and to fish out compounds from complex mixtures.

## Supplementary Material



## Notes

Chemical information and sources of 15 triterpenes; reported activity on RORγ and TGR5 of selected 15 triterpenes; compilation of the artificial microfractions (AMFs); AMF1–AMF4 2D HetCA pseudospectrum values; 2D HetCA results of microfractions MF33–MF35; 14 most active features from the MF33–MF35 2D HetCA; ^1^H NMR data of 2α,19α-dihydroxy-3-oxo-12-ursen-28-oic acid (**16**); parameters for ^13^C APT, ^1^H–^13^C HMBC, and ^1^H–^1^H COSY experiments; inverse agonist activity of selected 15 triterpenes on RORγ and TGR5; ^1^H NMR stack plot of compounds **1**, **7**, **8**, **12**, and **13**; ^1^H–^13^C HSQC spectrum of ursolic acid (**1**); ^1^H–^13^C HSQC spectrum of faradiol (**7**); ^1^H–^13^C HSQC spectrum of betulin (**8**); ^1^H–^13^C HSQC spectrum of hederagenin (**12**); ^1^H–^13^C HSQC spectrum of bayogenin (**13**); ^1^H NMR stack plot of AMF1–AMF4; ^1^H–^13^C HSQC spectrum of AMF1; ^1^H–^13^C HSQC spectrum of AMF2; ^1^H–^13^C HSQC spectrum of AMF3; ^1^H–^13^C HSQC spectrum of AMF4; inverse agonist activity of MF1–MF45 on RORγ; UHPSFC-ELSD results of MF33–MF35 and references **1** and **2**; UHPSFC-MS results of MF33–MF35 and references **1** and **2**; stacked ^1^H NMR plot of microfractions MF33–MF35; ^1^H–^13^C HSQC spectrum of MF33; ^1^H–^13^C HSQC spectrum of MF34; ^1^H–^13^C HSQC spectrum of MF35; UHPSFC-ELSD chromatograms of MF33, A3, and compounds **1** and **2**; overlayed ^1^H NMR of A3–A5 at δ_H_ 4.55; UHPSFC-ELSD results for A3, and B1–B3; stacked plot of the ^1^H NMR spectra of fractions B1–B3; bioactivity testing of compounds **16**, **17**, and **18** on RORγ; UHPSFC-ELSD results for EJD with integrated area under the curves (PDF)
