# Supplementary material for: Biochemometric 2D NMR-Based Heterocovariance Analysis: A Targeted Approach for Identifying Bioactive Compounds in Complex Mixtures
Source: Anal Chem. 2025 Oct 12;97(41):22508–17. doi: 10.1021/acs.analchem.5c02419 (PMC12547857; doi:10.1021/acs.analchem.5c02419)
Supplement: Supplementary file 1 [file ac5c02419_si_001.pdf]

SUPPORTING INFORMATION

Biochemometric 2D NMR-based heterocovariance analysis: A targeted approach for identifying bioactive compounds in complex mixtures

Sigrid Adelsberger,[†,§] Alexander F. Perhal,[†] Lorenza Bertaina,[†,§] Patrik F. Schwarz,[†,§]

Verena M. Dirsch,[†] Judith M. Rollinger,[†] Ulrike Grienke[,†]*

[†] Division of Pharmacognosy, Department of Pharmaceutical Sciences, Faculty of Life Sciences, University of Vienna, Josef-Holaubek-Platz 2, 1090 Vienna, Austria

[§] Vienna Doctoral School of Pharmaceutical, Nutritional and Sport Sciences, University of Vienna, Josef-Holaubek-Platz 2, 1090 Vienna, Austria

* Corresponding Author

Ulrike Grienke – Division of Pharmacognosy, Department of Pharmaceutical Sciences, Faculty of Life Sciences, University of Vienna, Josef-Holaubek-Platz 2, 1090 Vienna, Austria; orcid.org/0000-0003-0305-9270; E-mail: ulrike.grienke@univie.ac.at

TABLE OF CONTENTS

Table S1. Chemical information and sources of 15 triterpenes……..S-3
Table S2. Reported activity on RORγ and TGR5 of selected 15 triterpenes……..……………..S-4
Table S3. Compilation of the artificial microfractions (AMFs)………………………………S-5
Table S4. AMF1–AMF4 2D HetCA pseudospectrum values……………...………...………S-6
Table S5. 2D HetCA results of microfractions MF33–MF35………………..………………..…S-7
Table S6. 14 most active features from the MF33–MF35 2D HetCA.......………………..……..S-8
Table S7. ^{1}H NMR data of 2α,19α-dihydroxy-3-oxo-12-ursen-28-oic acid (**16**)……………..….......S-9
Table S8. Parameters for ^{13}C APT, ^{1}H-^{13}C HMBC, and ^{1}H-^{1}H COSY experiments.....………S-10
Figure S1. Inverse agonist activity of selected 15 triterpenes on RORγ and TGR5..…………S-11
Figure S2. ^{1}H NMR stack plot of compounds **1**, **7**, **8**, **12**, and **13**……….…………….…......S-12
Figure S3. ^{1}H–^{13}C HSQC spectrum of ursolic acid (**1**)……………………………….………S-13
Figure S4. ^{1}H–^{13}C HSQC spectrum of faradiol (**7**)………………………………….……….S-14
Figure S5. ^{1}H–^{13}C HSQC spectrum of betulin (**8**)……………………………………………S-15
Figure S6. ^{1}H–^{13}C HSQC spectrum of hederagenin (**12**)…………………….……………..…S-16
Figure S7. ^{1}H–^{13}C HSQC spectrum of bayogenin (**13**)…………………….…………….……S-17
Figure S8. ^{1}H NMR stack plot of AMF1–AMF4...……………………………………………S-18
Figure S9. ^{1}H−^{13}C HSQC spectrum of AMF1...……………………………………..….S-19
Figure S10. ^{1}H−^{13}C HSQC spectrum of AMF2...……………………………………..….S-20
Figure S11. ^{1}H−^{13}C HSQC spectrum of AMF3...……………………………………..….S-21
Figure S12. ^{1}H−^{13}C HSQC spectrum of AMF4...………..…………………………..…..….S-22
Figure S13. Inverse agonist activity of MF1–MF45 on RORγ……..………………………..….S-23
Figure S14. UHPSFC-ELSD results of MF33–MF35 and references **1** and **2**……………...….S-24
Figure S15. UHPSFC-MS results of MF33–MF35 and references **1** and **2**…………………...S-25
Figure S16. Stacked ^{1}H NMR plot of microfractions MF33–MF35……..…………………....S-26
Figure S17. ^{1}H–^{13}C HSQC spectrum of MF33. …………………….....……..……….…S-27
Figure S18. ^{1}H–^{13}C HSQC spectrum of MF34. …………………….....……..……….…S-28
Figure S19. ^{1}H–^{13}C HSQC spectrum of MF35. …………………….....……..……….…S-29
Figure S20. UHPSFC-ELSD chromatograms of MF33, A3, and compounds **1** and **2**…….......S-30
Figure S21. Overlayed ^{1}H NMR of A3–A5 at δ_{H} 4.55……………………………….…….…S-31
Figure S22. UHPSFC-ELSD results for A3, and B1–B3…………………………......….S-32
Figure S23. Stacked plot of the ^{1}H NMR spectra of fractions B1–B3……………...………..S-33
Figure S24. Bioactivity testing of compounds **16**, **17**, and **18** on RORγ………………………S-34

Figure S25. UHPSFC-ELSD results for EJD with integrated area under the curves…………………………………………………………………………………...……...S-35

Supporting Information References……………………………………….………….……......S-36

Table S1. Chemical information and sources of 15 triterpenes.

triterpene	sum formula	molecular weight	provider	batch number	puritiy [%]
ursolic acid (**1**)	$C_{30}H_{48}O_3$	456.70	PhytoLab	11781	99.99
oleanolic acid (**2**)	$C_{30}H_{48}O_3$	456.70	Sigma Aldrich	SLBD5617V	99.60
echinocystic acid (**3**)	$C_{30}H_{48}O_4$	472.70	PhytoLab	11854	99.72
erythrodiol (**4**)	$C_{30}H_{50}O_2$	442.72	PhytoLab	10531	99.50
uvaol (**5**)	$C_{30}H_{50}O_2$	442.72	isolated in house	/	98.05
α-amyrin (**6**)	$C_{30}H_{50}O$	426.72	PhytoLab	10429	99.54
faradiol (**7**)	$C_{30}H_{50}O_2$	442.72	PhytoLab	18231	99.36
betulin (**8**)	$C_{30}H_{50}O_2$	442.72	PhytoLab	19269	99.54
lupeol (**9**)	$C_{30}H_{50}O$	426.72	Carl Roth	3991247	99.99
maslinic acid (**10**)	$C_{30}H_{48}O_4$	472.70	PhytoLab	6695	99.91
corosolic acid (**11**)	$C_{30}H_{48}O_4$	472.70	PhytoLab	7053	99.71
hederagenin (**12**)	$C_{30}H_{48}O_4$	472.70	PhytoLab	12808	99.82
bayogenin (**13**)	$C_{30}H_{48}O_5$	488.70	PhytoLab	10392	99.95
asiatic acid (**14**)	$C_{30}H_{48}O_5$	488.70	PhytoLab	6265	99.14
ganoderic acid A (**15**)	$C_{30}H_{44}O_7$	516.67	PhytoLab	9512	98.00

The purity of uvaol (**5**) was evaluated via Ultra-high performance supercritical fluid chromatography (UHPSFC) using an evaporative light scattering detector (ELSD) on a Waters Acquity UPC² device with a 1-aminoanthracene column (3.0 x 100 mm, 1.7 μm) as stationary phase at 45°C. The mobile phase consisted of supercritical carbon dioxide and methanol as a co-solvent, the flowrate was 1 mL/min. 1–2 mg of each TT were dissolved in n-hexane:isopropanol (both VWR Chemicals, Radnor, USA) 7:3. Areas under the curve were summed up to 100% and the purity was calculated as the percentage of the respective TT peak. A Waters Acquity Quadrupole Dalton (QDa) detector was used to evaluate the mass of the TTs. Methanol (VWR Chemicals, Radnor, USA) and double distilled water (Milli-Q-Plus ultrapure water device by Millipore Corporation, Bedford, MA, USA) 95:5 with 10 mM ammonium formate improved the ionization process. Waters software Empower 3 was used for data processing.

Table S2. Reported activity on RORγ and TGR5 of selected 15 triterpenes.

N/A means that no information is available. TTs such as **1**,[1] **2**,[1] **5**,[1] **11**,[2] **12**,[3] and to some extent also **14**,[2] are known as inverse agonists on RORγ and therefore decrease the transcriptional activity below the basal level,[1] while **10**[2] shows no activity on the same target. Also, there are studies that confirm TTs, such as **1**,[4, 5] **2**,[4, 6] **10**,[7] and **11**,[7] to be active on TGR 5, while compounds **6**,[4] **8**,[4] **12**,[8] **13**,[8] and **14**,[4] are reported to be inactive or show weak activity.

**Triterpene**	**Target(s)**	**Comment(s)**
ursolic acid (**1**)	RORγt antagonist[9] / inverse agonist[1, 10]	IC_{50} = 0.68±0.1 mM<br>co-activator binding to RORγt ligand binding domain (LBD) in TR-FRET assay[1]
	TGR5 agonist[4, 5]	EC_{50} = 1.43 μM, TGR5 luciferase assay[4]
oleanolic acid (**2**)	RORγ(t) inverse agonist[1]	EC_{50} = 8.589 μM<br>RORγ-LBD:Gal4-DNA binding domain (DBD) luciferase reporter assay in Jurkat cells[1]
	TGR5 agonist[4, 6]	EC_{50} = 2.25 μM, TGR5 luciferase assay[4]<br>EC_{50} = 1.42 μM, human TGR5 luciferase assay[6]
echinocystic acid (**3**)	RORγ(t) inverse agonist[11]	Best-fit IC_{50} = 2.127 μM<br>RORγ-Gal4 luciferase assay[11]
erythrodiol (**4**)	RORγ(t) inverse agonist[11]	Best-fit IC_{50} = 0.456 μM<br>RORγ-Gal4 luciferase assay[11]
uvaol (**5**)	RORγ(t) inverse agonist[1]	EC_{50} = 4.254 μM<br>RORγ-LBD:Gal4-DBD luciferase reporter assay in Jurkat cells[1, 12]
α-amyrin (**6**)	TGR5 agonist[4]	EC_{50} > 10 μM<br>TGR5 luciferase assay[4]
faradiol (**7**)	N/A	N/A
betulin (**8**)	TGR5 agonist[4]	EC_{50} > 10 μM, TGR5 luciferase assay[4]
lupeol (**9**)	N/A	N/A
maslinic acid (**10**)	no RORγ(t) activity[10]	GAL-RORγ-LBD reporter assay
	TGR5 agonist[7]	3.7 ± 0.7 μM, TGR5 luciferase reporter gene assay[7]
corosolic acid (**11**)	RORγ(t) inverse agonist[10]	EC = 2.52 μM, GAL-RORγ-LBD reporter assay[10]
	TGR5 agonist[7]	0.5 ± 1.0 μM, TGR5 luciferase reporter gene assay[7]
hederagenin (**12**)	reduced RORγ(t) activity[13]	Autoimmune encephalomyelitis (EAE) mouse model
	no TGR5 activity[8]	reporter gene-based luciferase assay in HEK 293T cells
bayogenin (**13**)	no TGR5 activity[8]	reporter gene-based luciferase assay in HEK 293T cells
asiatic acid (**14**)	RORγ(t) inverse agonist[10]	EC > 15 μM<br>GAL-RORγ-LBD reporter assay[10]
	TGR5 agonist[4]	EC_{50} > 10 μM<br>TGR5 luciferase assay[4]
ganoderic acid A (**15**)	N/A	N/A

Table S3. Compilation of the artificial microfractions (AMFs).

triterpene	ratios							
	AMF1	AMF2	AMF3	AMF4	AMF5	AMF6	AMF7	AMF8
ursolic acid (**1**)	3.50	1.00	0.50	0	0	0	0	0
hederagenin (**12**)	0	0	0.25	0.25	1.00	3.00	1.00	0.75
faradiol (**7**)	1.00	1.50	2.00	1.00	0.75	0	0	0
betulin (**8**)	1.50	3.50	5.00	6.00	5.00	3.50	2.00	1.50
bayogenin (**13**)	0	0	0	0	0.25	0.75	3.00	3.50

Table S4. AMF1–AMF4 2D HetCA pseudospectrum values.

Value pairs of δ_H,δ_C for each cross-peak with the same color-code as the correlation coefficient, with assignments to compounds **1**, **7**, **8**, and **12**.

δ_H (ppm)	δ_C (ppm)	δ_H (ppm)	δ_C (ppm)	δ_H (ppm)	δ_C (ppm)	δ_H (ppm)	δ_C (ppm)
5.31	118.1 (7)	2.25	29.2 (12)	1.56	33.9 (12)	1.00	38.5 (1)
5.30	122.4 (12)	2.19	52.5 (1)	1.52	47.3 (1)	0.98	14.9 (8)
5.25	125.7 (1)	2.16	39.7 (12)	1.51	30.5 (1)	0.97	49.5 (12)
4.68	109.8 (8)	2.15	21.1 (12)	1.50	32.7 (1)	0.97	28.1 (8)
4.58	109.8 (8)	2.10	37.2 (7)	1.33	38.8 (1)	0.95	20.9 (1)
3.80	60.7 (8)	2.02	51.8 (12)	1.33	36.7 (7)	0.94	16.9 (12)
3.73	71.2 (12)	2.01	39.7 (12)	1.29	33.9 (12)	0.93	15.2 (1)
3.68	75.9 (12)	1.93	29.2 (8)	1.21	29.3 (8)	0.90	23.4 (12)
3.44	76.1 (7)	1.77	29.2 (12)	1.21	18.7 (12)	0.87	11.1 (12)
3.43	71.2 (12)	1.74	44.6 (12)	1.09	27.8 (1)	0.82	16.1 (8)
3.33	60.7 (8)	1.68	19.2 (8)	1.08	23.3 (1)	0.76	15.1 (8)
3.18	79.1 (8)	1.67	23.9 (1)	1.04	47.3 (7)	0.72	55.0 (1)
2.38	47.9 (8)	1.60	48.9 (8)	1.02	16.3 (8)		

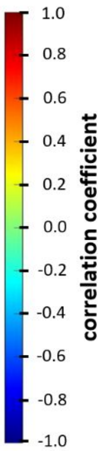

Table S5. 2D HetCA results of microfractions MF33–35.

The results for the microfractions (MFs) MF33–35 are given as pairs of δ_H,δ_C, together with the respective correlation coefficient (cc). The color-code of the cc, from dark blue with -1.00 to dark red with 1.00, is used for the font color of the δ_H,δ_C values.

δ_H (ppm)	δ_C (ppm)	cc	δ_H (ppm)	δ_C (ppm)	cc	δ_H (ppm)	δ_C (ppm)	cc	δ_H (ppm)	δ_C (ppm)	cc
5.36	128.9	0.995	1.70	36.9	-0.985	1.29	33.8	-0.922	1.02	18.8	0.986
5.29	122.3	0.869	1.70	23.9	-0.905	1.27	41.8	-0.922	1.01	27.5	0.737
5.28	125.7	-0.916	1.69	37.6	-0.993	1.27	22.7	-0.949	1.00	38.5	-0.879
5.28	122.7	0.957	1.69	26.0	-1.000	1.27	16.0	0.984	0.99	28.9	0.954
5.27	125.7	0.998	1.68	47.2	0.955	1.26	32.2	-0.957	0.99	27.9	0.938
5.25	125.7	0.978	1.68	41.8	-0.992	1.26	24.6	0.942	0.99	18.8	0.937
5.14	125.2	0.882	1.68	26.0	-0.979	1.25	29.5	-0.999	0.98	38.4	-0.947
4.55	69.3	0.992	1.67	36.5	-0.973	1.25	26.6	-0.931	0.98	28.0	0.957
4.00	66.4	0.955	1.67	29.3	0.906	1.24	41.8	-0.999	0.98	24.4	0.967
4.00	66.3	0.979	1.66	24.0	-0.984	1.24	37.5	0.998	0.95	41.0	0.983
3.90	76.3	0.553	1.65	38.4	-0.975	1.24	24.5	-0.967	0.95	38.8	0.923
3.79	63.2	-0.891	1.64	47.2	0.970	1.21	41.6	0.976	0.95	21.0	0.956
3.60	53.3	-0.977	1.63	41.6	-0.962	1.21	27.5	-0.978	0.95	20.9	0.955
3.60	51.5	0.973	1.62	27.5	-0.966	1.21	27.3	0.973	0.95	16.1	0.980
3.49	75.9	0.770	1.62	24.8	-0.938	1.21	17.0	0.990	0.95	13.7	0.989
3.43	78.8	0.829	1.60	38.4	-0.946	1.20	48.0	0.969	0.93	38.6	-0.985
3.22	78.9	0.934	1.60	37.7	-0.997	1.19	57.8	0.925	0.93	23.6	0.953
3.21	78.9	0.957	1.60	32.4	-0.950	1.19	48.0	0.927	0.93	21.5	0.941
2.82	41.0	0.985	1.61	25.2	-0.982	1.19	19.3	1.000	0.93	15.2	0.985
2.80	40.0	0.983	1.59	25.4	-0.995	1.18	18.0	0.995	0.92	45.9	0.986
2.60	53.1	0.925	1.58	37.5	-0.913	1.17	26.2	0.970	0.92	45.7	0.990
2.55	53.0	0.970	1.58	32.3	-0.961	1.17	24.6	0.770	0.92	23.5	0.958
2.54	25.4	-0.977	1.58	25.4	-1.000	1.17	23.0	0.874	0.92	18.7	0.932
2.41	46.7	1.000	1.56	20.7	-0.996	1.17	16.2	-0.997	0.91	15.2	0.908
2.40	49.6	-0.838	1.54	47.6	0.940	1.16	24.8	0.984	0.91	23.8	0.790
2.35	33.6	-0.909	1.53	30.6	-0.972	1.15	49.7	0.765	0.90	33.0	0.953
2.30	34.5	-0.940	1.53	32.5	-1.000	1.15	18.4	-0.938	0.89	14.2	0.965
2.19	52.5	0.985	1.53	19.3	-0.905	1.14	39.4	-0.997	0.86	39.0	1.000
2.18	52.5	0.962	1.52	47.4	0.977	1.14	26.0	0.935	0.86	21.9	0.989
2.18	30.3	0.934	1.52	18.2	-0.963	1.14	17.4	0.895	0.86	17.0	0.967
2.13	28.3	0.856	1.51	30.6	-0.967	1.11	27.7	0.975	0.86	16.9	0.967
2.07	23.9	-0.997	1.50	18.2	-0.986	1.11	23.0	-0.945	0.86	16.7	0.967
2.02	23.6	-0.993	1.48	32.8	-0.980	1.11	21.7	0.979	0.85	21.7	0.977
1.99	23.8	-0.997	1.47	25.0	-0.993	1.11	21.5	0.803	0.85	19.7	0.988
1.98	27.6	-0.358	1.47	18.0	-1.000	1.10	27.8	-0.982	0.85	16.6	0.961
1.95	23.4	-0.982	1.45	18.0	-1.000	1.10	16.6	0.999	0.85	11.9	0.963
1.92	23.0	-0.967	1.42	41.2	0.974	1.09	23.6	0.978	0.84	19.7	0.988
1.89	18.6	0.610	1.38	32.5	-0.562	1.08	27.9	0.969	0.83	19.7	0.958
1.87	27.8	-0.920	1.36	18.2	-1.000	1.08	23.3	-0.966	0.81	17.5	0.914
1.85	27.9	-0.965	1.34	33.6	-0.963	1.08	16.2	0.989	0.80	17.1	0.958
1.81	37.6	-0.962	1.34	32.7	-0.989	1.07	27.3	-0.890	0.79	18.9	-0.354
1.77	32.3	-0.935	1.33	38.8	0.970	1.07	17.0	0.992	0.79	17.0	0.977
1.75	37.3	-1.000	1.33	25.2	-0.955	1.06	37.5	-0.999	0.78	15.3	0.918
1.75	27.3	-0.990	1.33	17.9	-0.999	1.06	18.6	0.983	0.77	15.6	0.967
1.74	46.8	0.938	1.32	38.9	0.974	1.05	21.1	0.947	0.77	15.4	0.967
1.74	28.2	-0.973	1.32	33.0	-0.986	1.04	18.5	0.933	0.75	17.0	0.972
1.74	23.8	-0.994	1.30	32.3	-0.996	1.04	16.6	0.970	0.73	55.2	0.961
1.72	36.6	-0.999	1.30	29.1	-0.935	1.03	28.2	0.998	0.72	55.0	0.977
1.72	36.5	-0.995	1.30	26.0	-0.999	1.02	39.0	0.958	0.70	12.1	0.974
1.71	27.3	-0.939	1.30	18.8	0.526	1.02	28.4	0.990	0.67	16.6	0.946
1.70	47.1	1.000	1.29	37.3	-1.000	1.02	24.4	0.716			

Table S6. 14 most active features from the MF33–MF35 2D HetCA.

The HSQC-based data sets were calculated for RORγ activity of MF33–35, listed as pairs of δ_H,δ_C, with the corresponding correlation coefficient (cc).

δ_H (ppm)	δ_C (ppm)	cc
2.41	46.7	1.0000
1.70	47.1	0.9999
0.86	39.0	0.9998
1.19	57.8	0.9996
1.10	16.6	0.9989
1.03	28.2	0.9984
1.24	24.5	0.9983
5.27	125.7	0.9981
1.18	18.0	0.9951
5.36	128.9	0.9950
1.07	17.0	0.9919
4.55	69.3	0.9916
1.21	17.0	0.9904
1.02	28.4	0.9903

Table S7. ^{1}H NMR data of 2α,19α-dihydroxy-3-oxo-12-ursen-28-oic acid (**16**).

The measurements were performed in deuterated chloroform-D1, at 500.19 MHz for ^{1}H NMR and 125.77 MHz for ^{13}C NMR.

pos	∫H	multiplicity	δH	δC	C	δC HMBC							δH COSY
1	1.74	dd, J (6.58/12.53)	1.15, 2.40	49.6	CH2	16.0	37.8	57.8	69.3	216.8			
2	0.98	dd, J (6.57/12.60)	4.55	69.3	CH	49.6	216.8						1.15, 2.40
3				216.8	C								
4				47.8	C								
5		overlapped	1.19	57.8	CH								
6		overlapped	1.19, 1.53	19.3	CH2								
7		overlapped	1.38, 1.53	32.5	CH2								
8				41.4	C								
9		overlapped	1.70	47.1	CH								
10				37.8	C								
11	2.98	dd, J (3.41/8.97)	1.70, 2.07	23.9	CH2	40.2	47.1	128.9	138.3				
12	1.00	t, J (3.47)	5.36	128.9	CH	23.9	41.4	47.1	53.0	73.2			1.70, 2.07
13				138.3	C								
14				40.2	C								
15		overlapped	1.03, 1.74	28.2	CH2								
16		overlapped	1.59, 2.54	25.4	CH2								
17				47.9	C								
18		overlapped	2.55	53.0	CH	25.4	41.2	47.9	73.2	128.9	138.3	182.4	1.05, 1.74, 1.59
19				73.2	C								
20		overlapped	1.42	41.2	CH								
21		overlapped	1.30, 1.69	26.0	CH2								
22		overlapped	1.69, 1.81	37.6	CH2								
23	2.98	s	1.16	24.8	CH3	21.7	47.8	57.8	216.8				
24	2.95	s	1.11	21.7	CH3	24.8	47.8	57.8	216.8				
25	2.91	s	1.27	16.0	CH3	37.8	47.1	49.6	57.8				
26	2.76	s	0.80	17.1	CH3	32.5	40.2	41.4	47.1				
27	3.02	s	1.24	24.5	CH3	28.2	40.2	41.4	138.3				
28				182.4	C								
29	2.79	s	1.21	27.5	CH3	41.2	53.0	73.2					
30	3.38	d, J (6.70)	0.95	16.1	CH3	26.0	41.2	73.2					

Table S8. Parameters for ^{13}C APT, ^{1}H-^{13}C HMBC, and ^{1}H-^{1}H COSY experiments.

For structure elucidation of B1– B3, additionally to ^{1}H NMR and ^{1}H–^{13}C HSQC, also ^{13}C APT, ^{1}H-^{13}C HMBC, and ^{1}H-^{1}H COSY experiments were conducted using standard Bruker pulse sequence programs.

NMR experiment	Pulse sequence and parameters
^{13}C APT	jmod<br>D1 = 3s (in addition to the acquisition time of 0.865 s),<br>SW = 37878.788 Hz, SI = 64k, 4096 scans, total time ~4.4 h
^{1}H-^{13}C HMBC	hmbcgpndqf<br>D1 = 3s (in addition to 0.2 s acquisition time), 2 scans, SW = 10kHz for ^{1}H and SW = 37.7 kHz for ^{13}C, SI = 4k for ^{1}H and SI = 256 for ^{13}C, total time ~1.8 h
^{1}H-^{1}H COSY	cosyqf45<br>D1 = 3s (in addition to 0.2 s of acquisition time), 4 scans, SI = 1K and SW = 5151 Hz in both dimensions, total time ~3.6 h

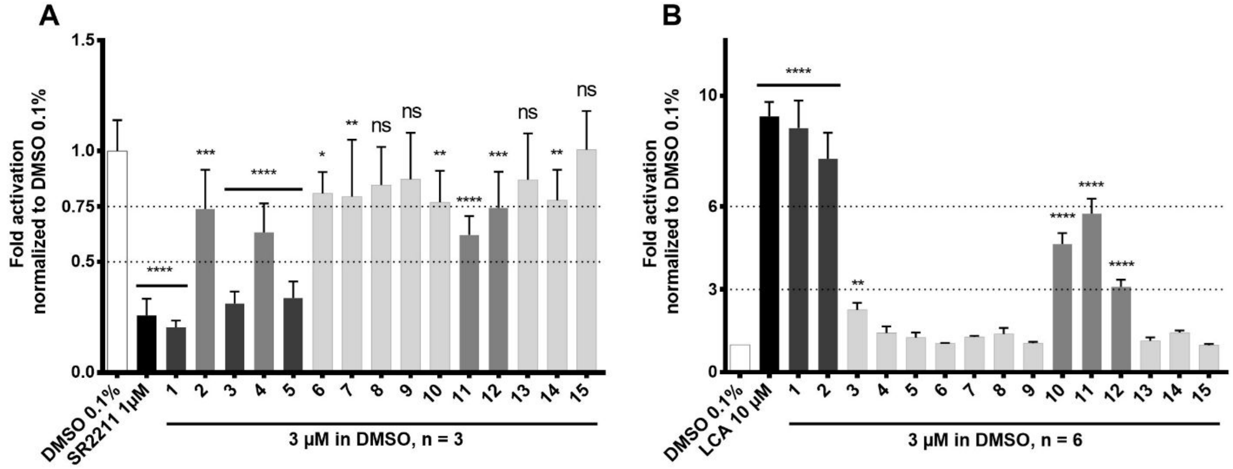

Figure S1. Activity of selected 15 triterpenes on ROR*γ* and TGR5.

(A) The inverse agonist activity on ROR*γ* in ROR*γ*-Gal4 luciferase assays was compared to DMSO 0.1% as vehicle control, with SR2211 as positive control. (B) Agonistic activity on TGR5, compared to DMSO 0.1% as vehicle control, with 10 *μ*M lithocholic acid (LCA) as positive control in CRE-luciferase assays (B) of 15 selected TTs (3 μM). Results are expressed as fold activations, bars represent transactivation activities expressed as means ± standard deviation of n biological replicates measured in technical quadruplicates. One-way ANOVA followed by Dunnett's post hoc test were used for statistical analysis. **** $p \leq 0.0001$, *** $p \leq 0.001$, ** $p \leq 0.01$, * $p \leq 0.05$ compared to vehicle control. For ROR*γ* inverse agonists, <0.5-fold activation was considered as active, 0.5–0.75-fold activation was classified as moderately active, and from >0.75-fold activation, compounds were considered inactive. For TGR5 agonists, >6-fold activation was classified as active, 3–6-fold as moderately active, and <3-fold as inactive. Bars of active compounds are marked in dark grey, of moderately active compounds in medium grey, and of inactive compounds marked in light grey.

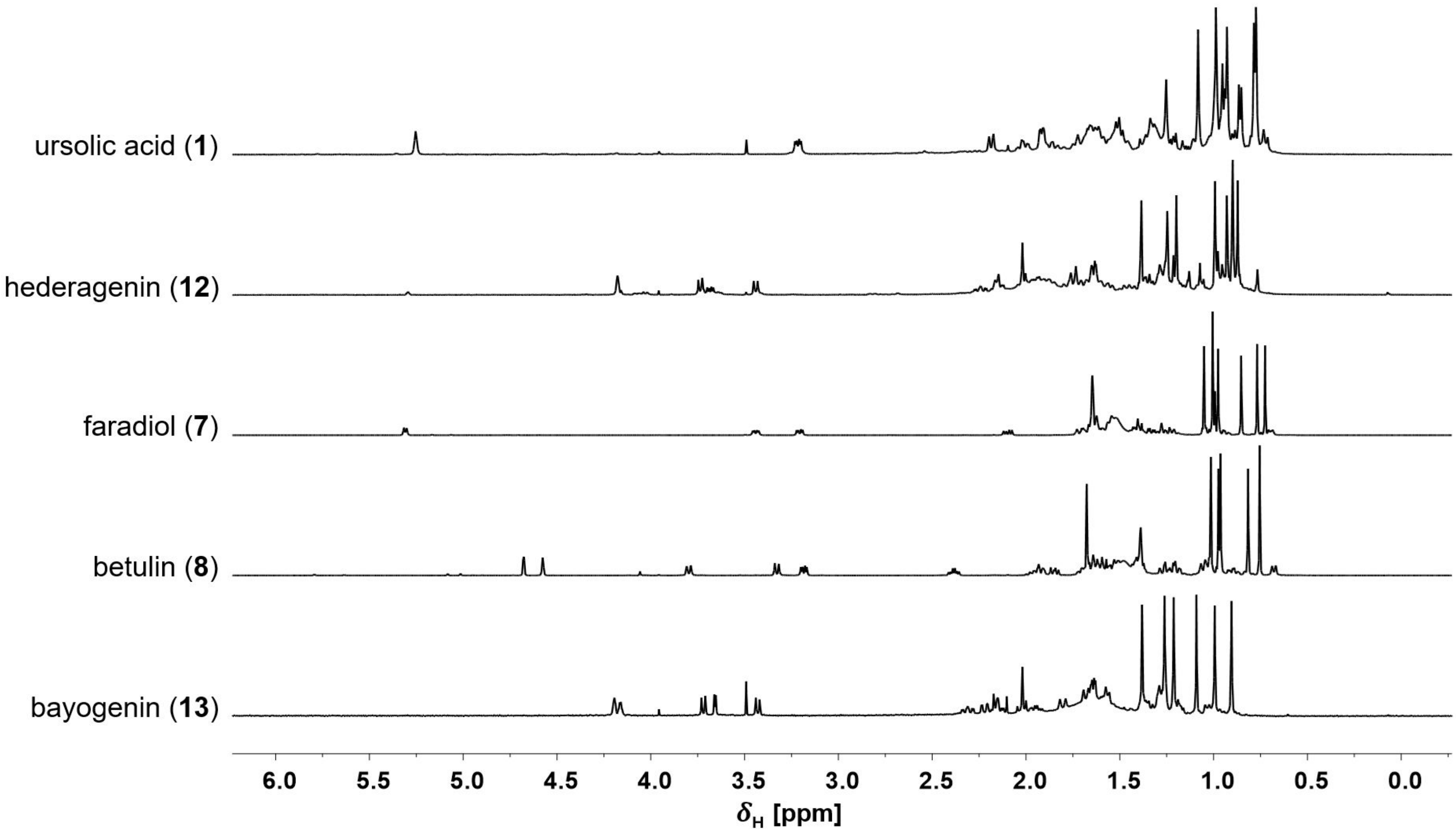

Figure S2. ^{1}H NMR stack plot of pure compounds **1**, **7**, **8**, **12**, and **13**.

The measurement was performed at 500.19 MHz in deuterated chloroform-D1 at 500.19 MHz.

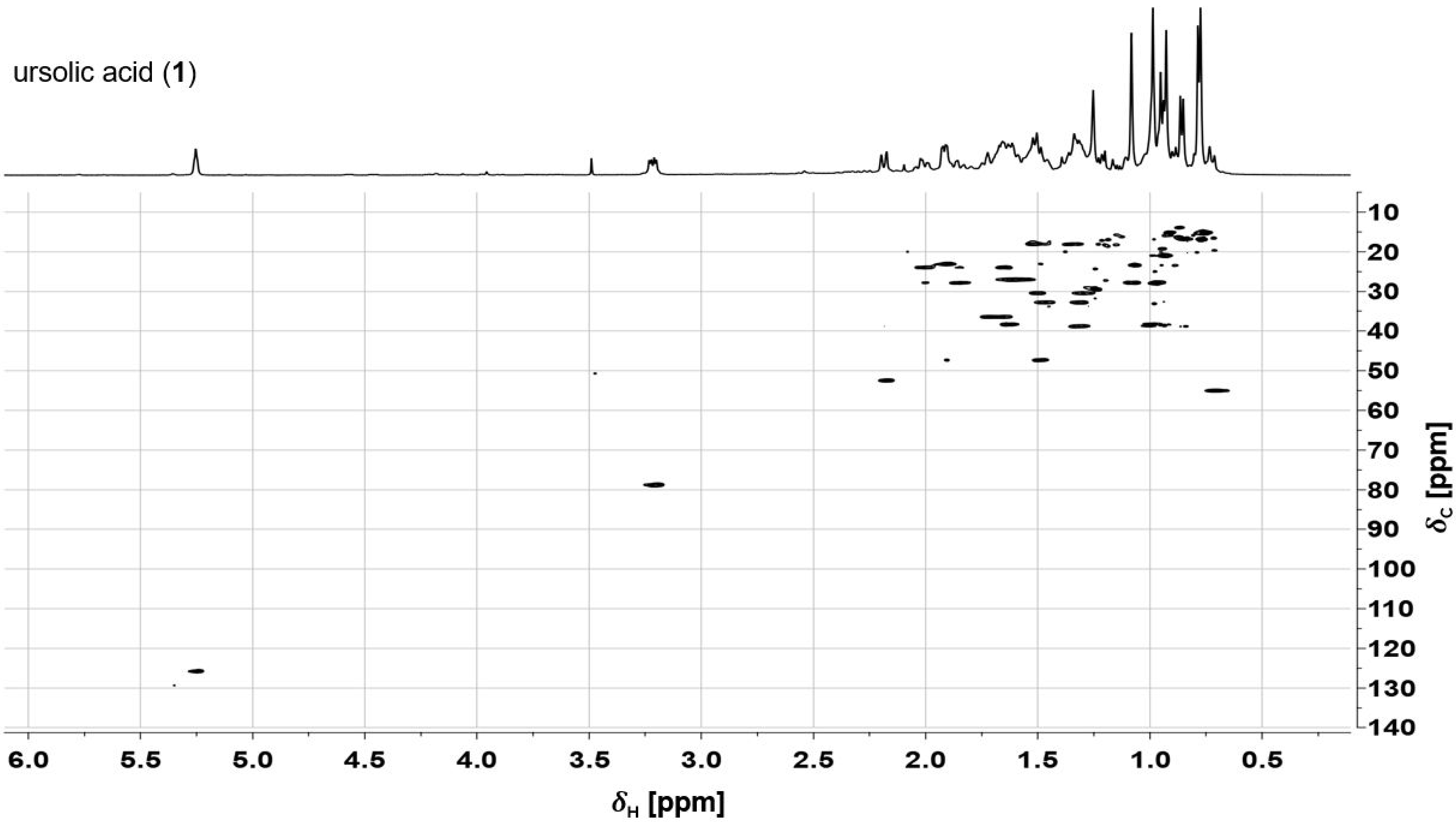

Figure S3. ^{1}H−^{13}C HSQC spectrum of ursolic acid (**1**).

The measurement was performed in deuterated chloroform-D1 at 500.19 MHz for ^{1}H NMR and 125.77 MHz for ^{13}C NMR.

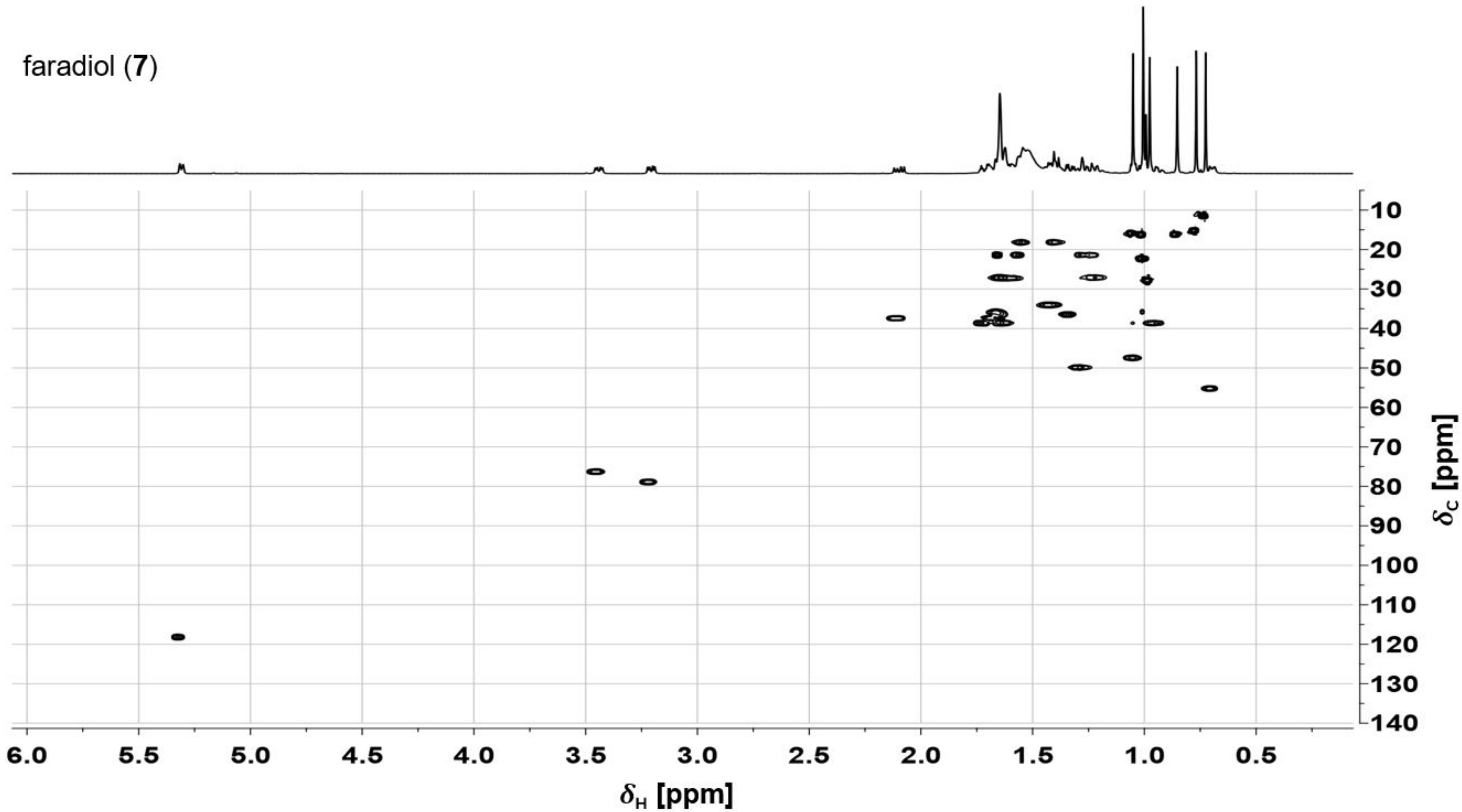

Figure S4. $^{1}H-^{13}C$ HSQC spectrum of faradiol (**7**).

The measurement was performed in deuterated chloroform-D1 at 500.19 MHz for ^{1}H NMR and 125.77 MHz for ^{13}C NMR.

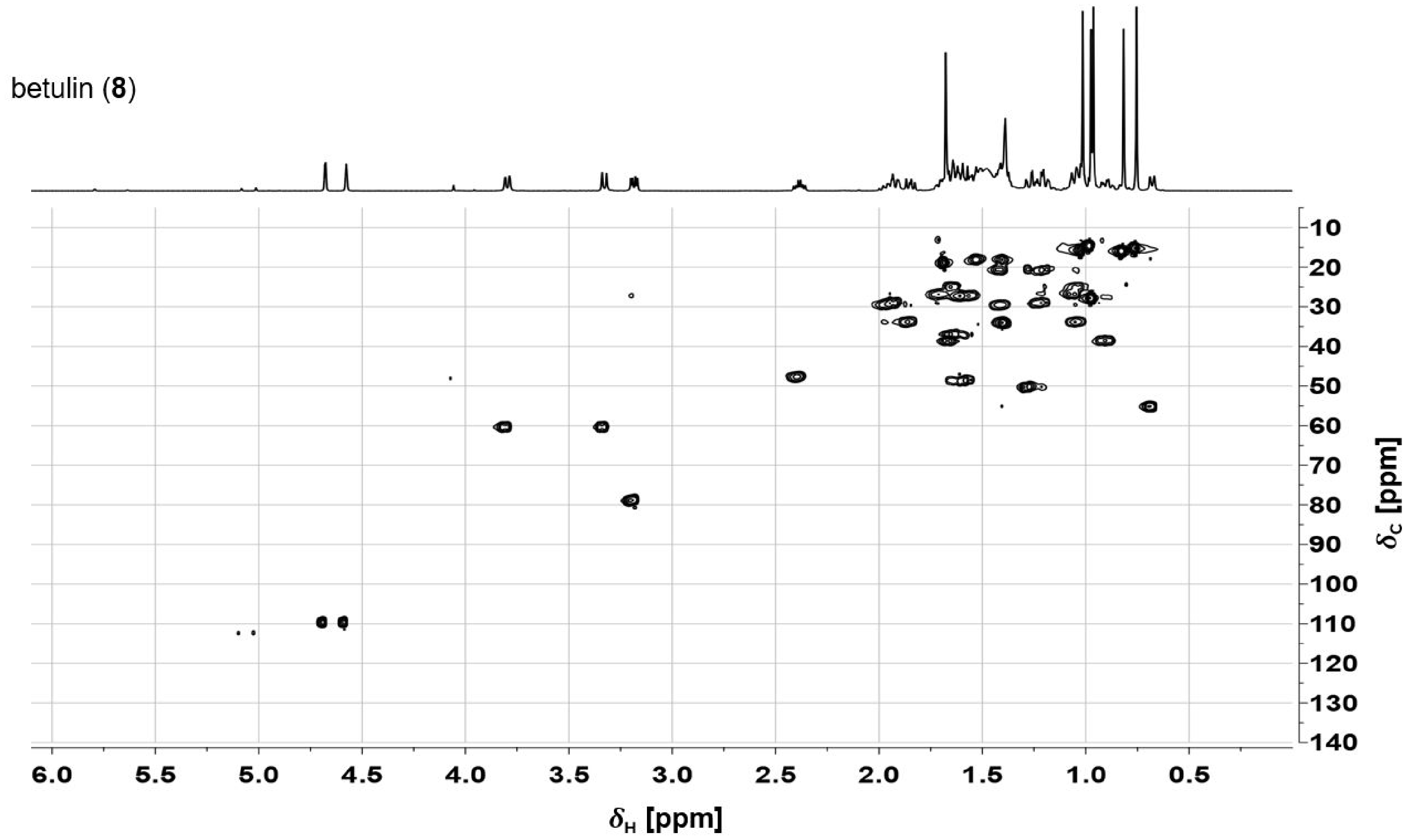

Figure S5. $^{1}H-^{13}C$ HSQC spectrum of betulin (**8**).

The measurement was performed in deuterated chloroform-D1 at 500.19 MHz for ^{1}H NMR and 125.77 MHz for ^{13}C NMR.

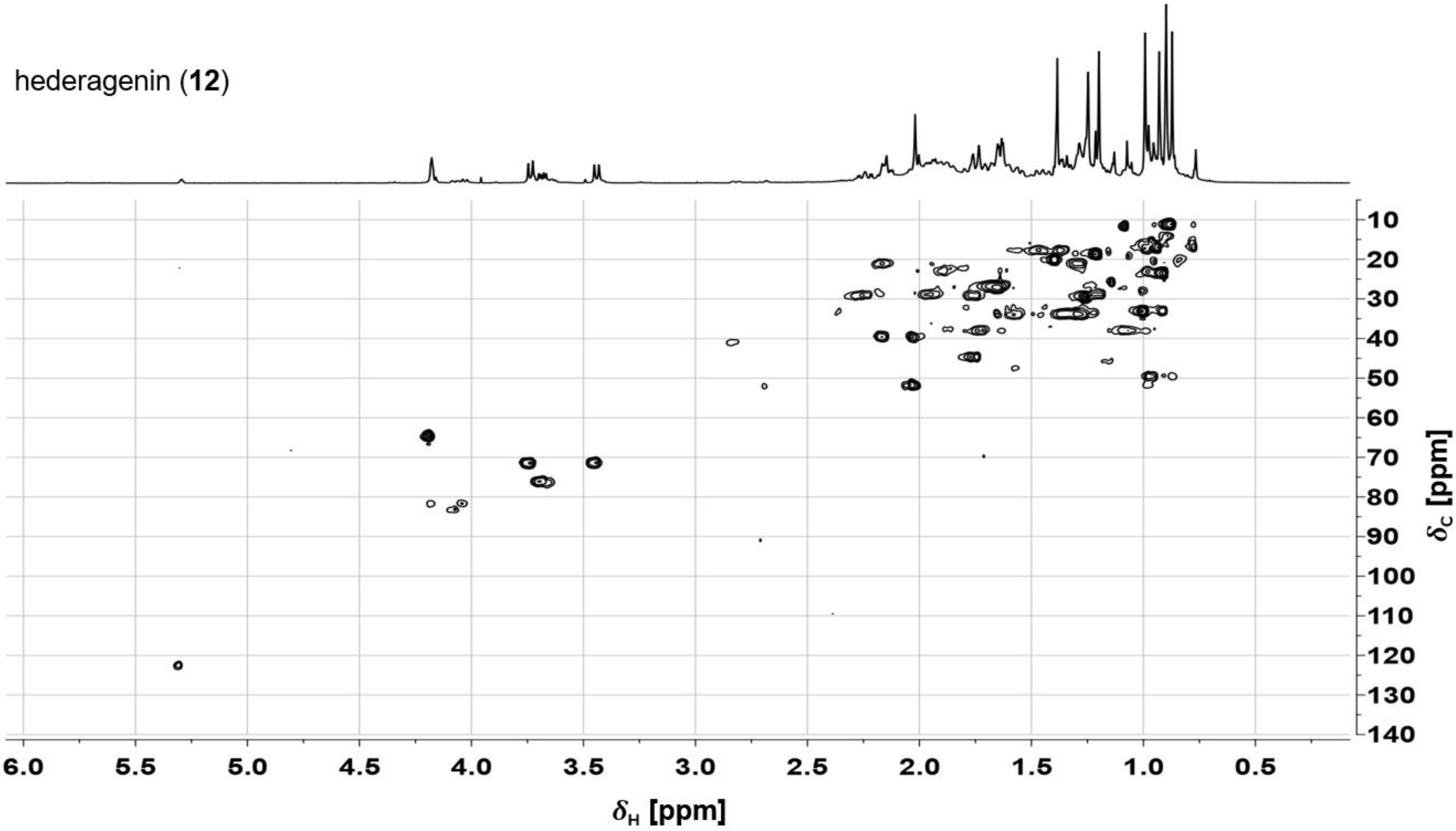

Figure S6. $^{1}H-^{13}C$ HSQC spectrum of hederagenin (**12**).

The measurement was performed in deuterated chloroform-D1 at 500.19 MHz for ^{1}H NMR and 125.77 MHz for ^{13}C NMR.

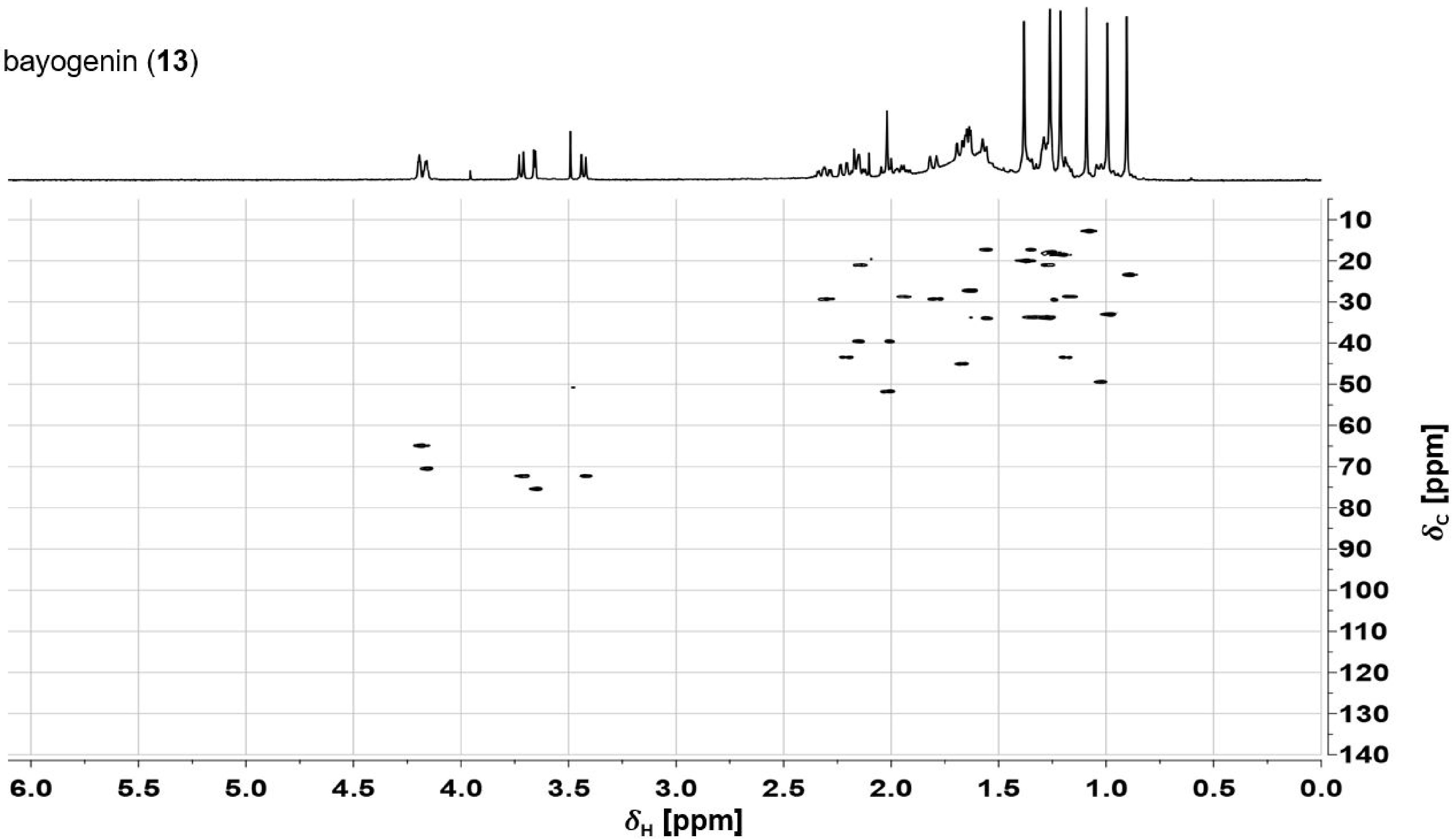

Figure S7. ^{1}H−^{13}C HSQC spectrum of bayogenin (**13**).

The measurement was performed in deuterated chloroform-D1 at 500.19 MHz for ^{1}H NMR and 125.77 MHz for ^{13}C NMR.

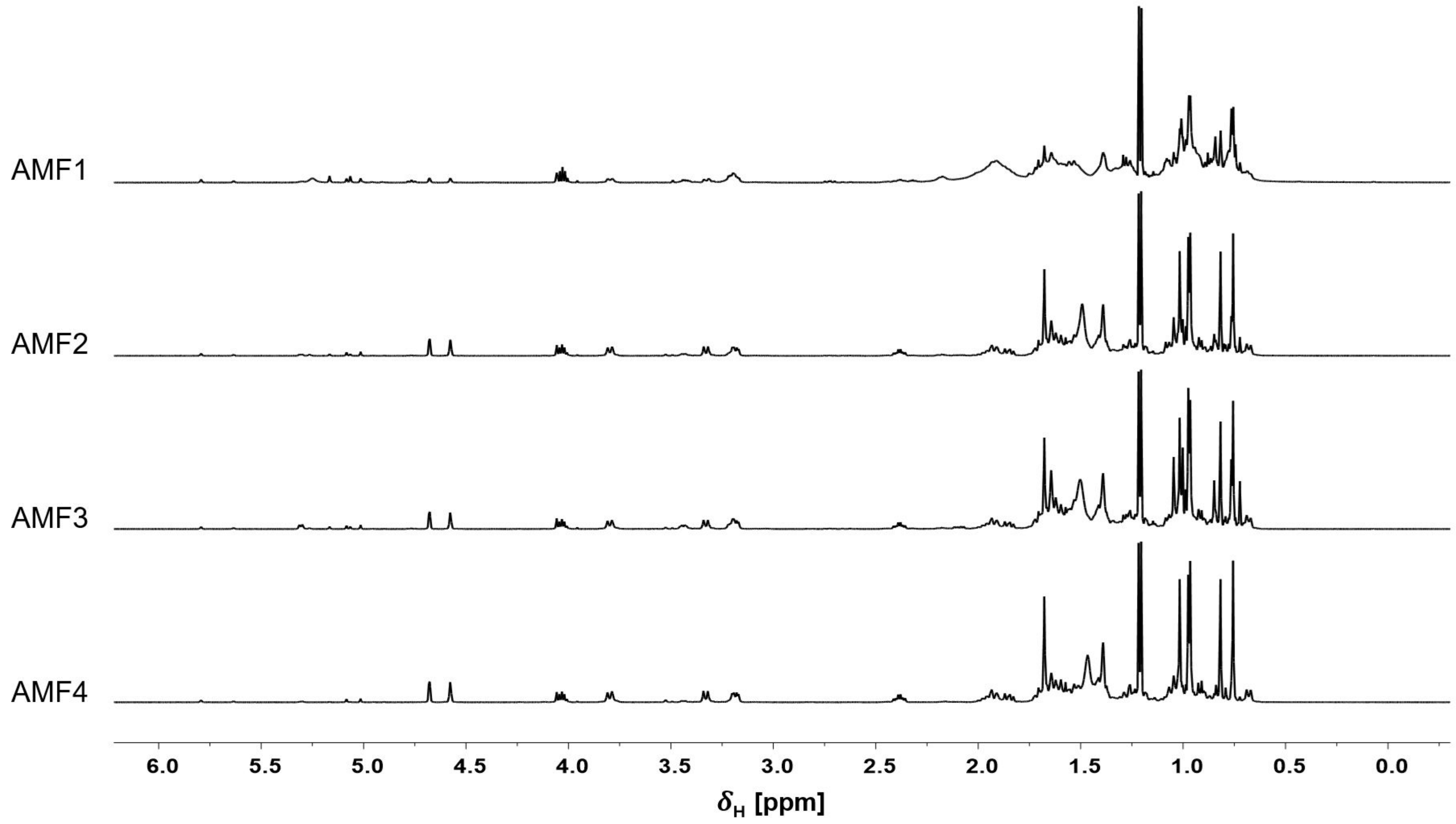

Figure S8. ^{1}H NMR stack plot of AMF1–AMF4.

The measurements were performed in deuterated chloroform-D1 at 500.19 MHz. All samples contain varying concentrations of the pure compounds ursolic acid (**1**), hederagenin (**12**), faradiol (**7**), and betulin (**8**).

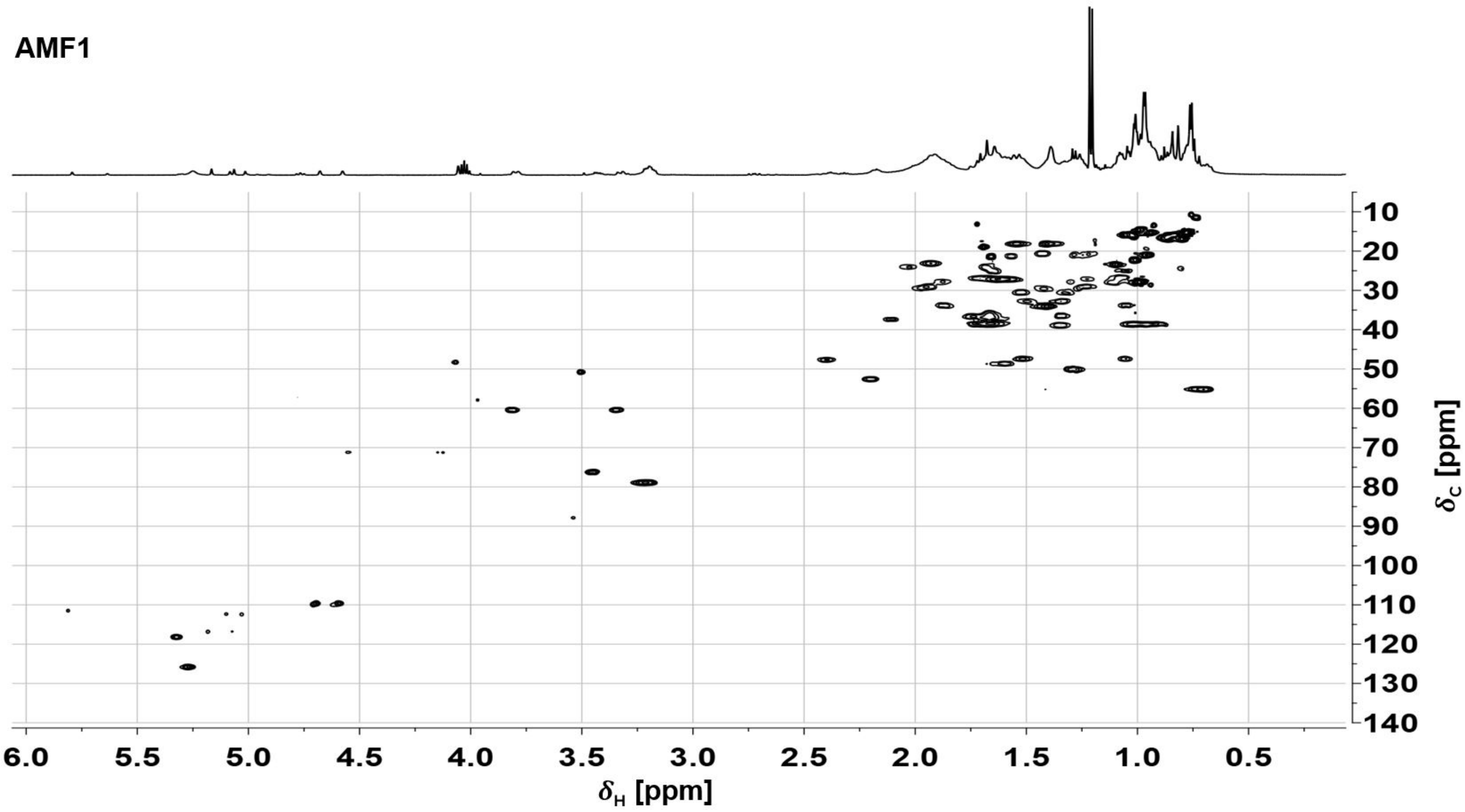

Figure S9. $^{1}H-^{13}C$ HSQC spectrum of AMF1.

The measurement was performed in deuterated chloroform-D1 at 500.19 MHz for ^{1}H NMR and 125.77 MHz for ^{13}C NMR.

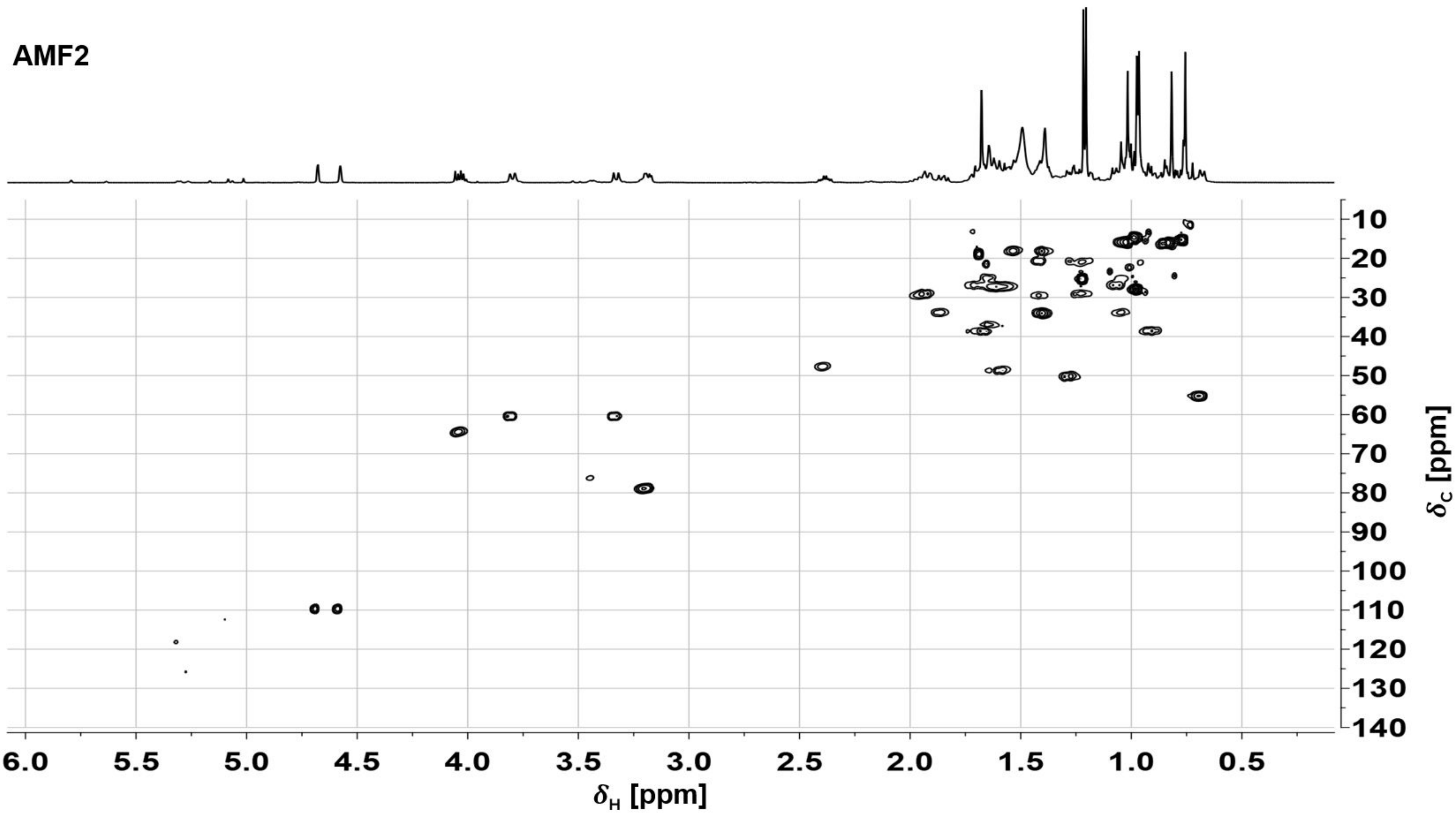

Figure S10. $^{1}H-^{13}C$ HSQC spectrum of AMF2.

The measurement was performed in deuterated chloroform-D1 at 500.19 MHz for ^{1}H NMR and 125.77 MHz for ^{13}C NMR.

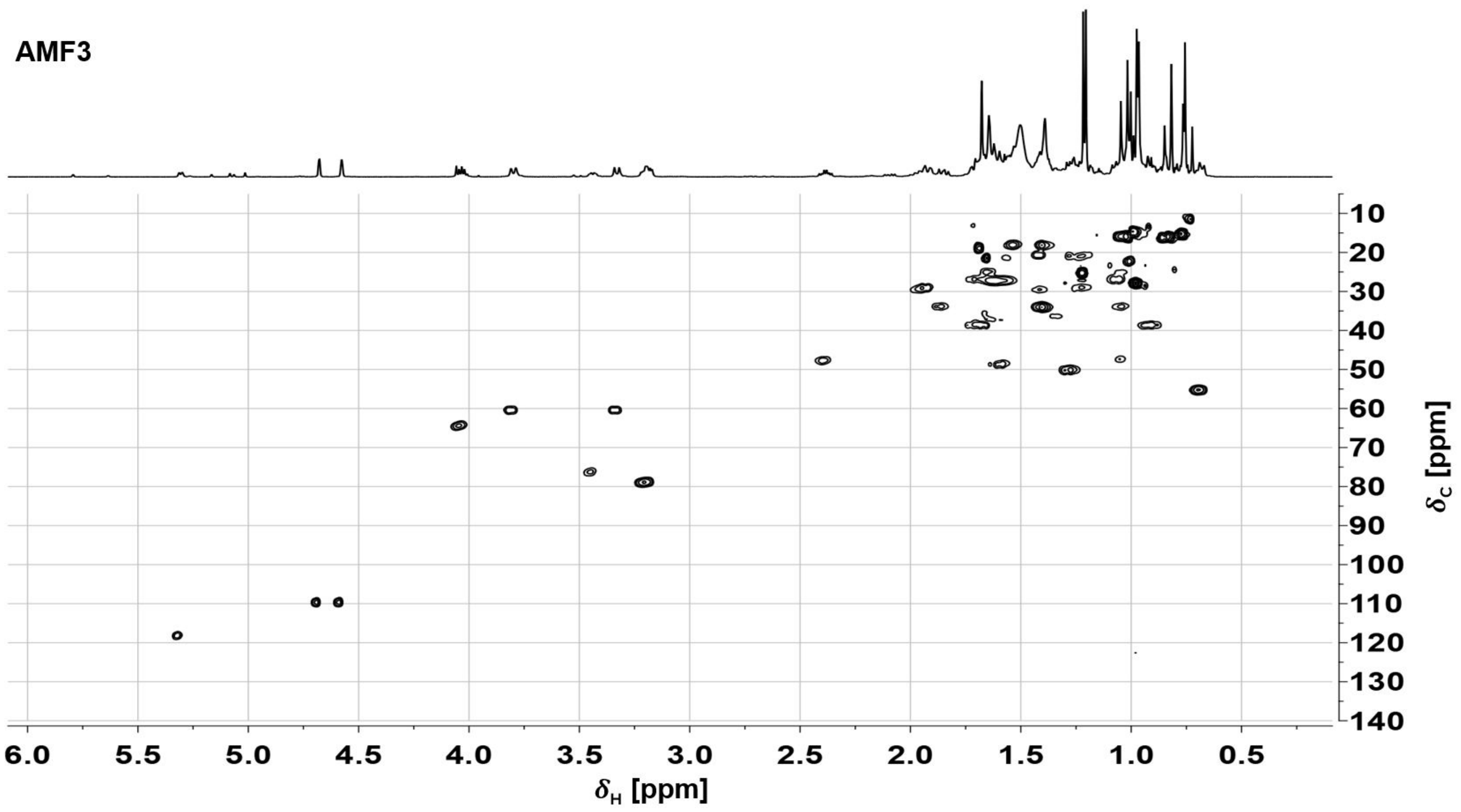

Figure S11. $^{1}H-^{13}C$ HSQC spectrum of AMF3.

The measurement was performed in deuterated chloroform-D1 at 500.19 MHz for ^{1}H NMR and 125.77 MHz for ^{13}C NMR.

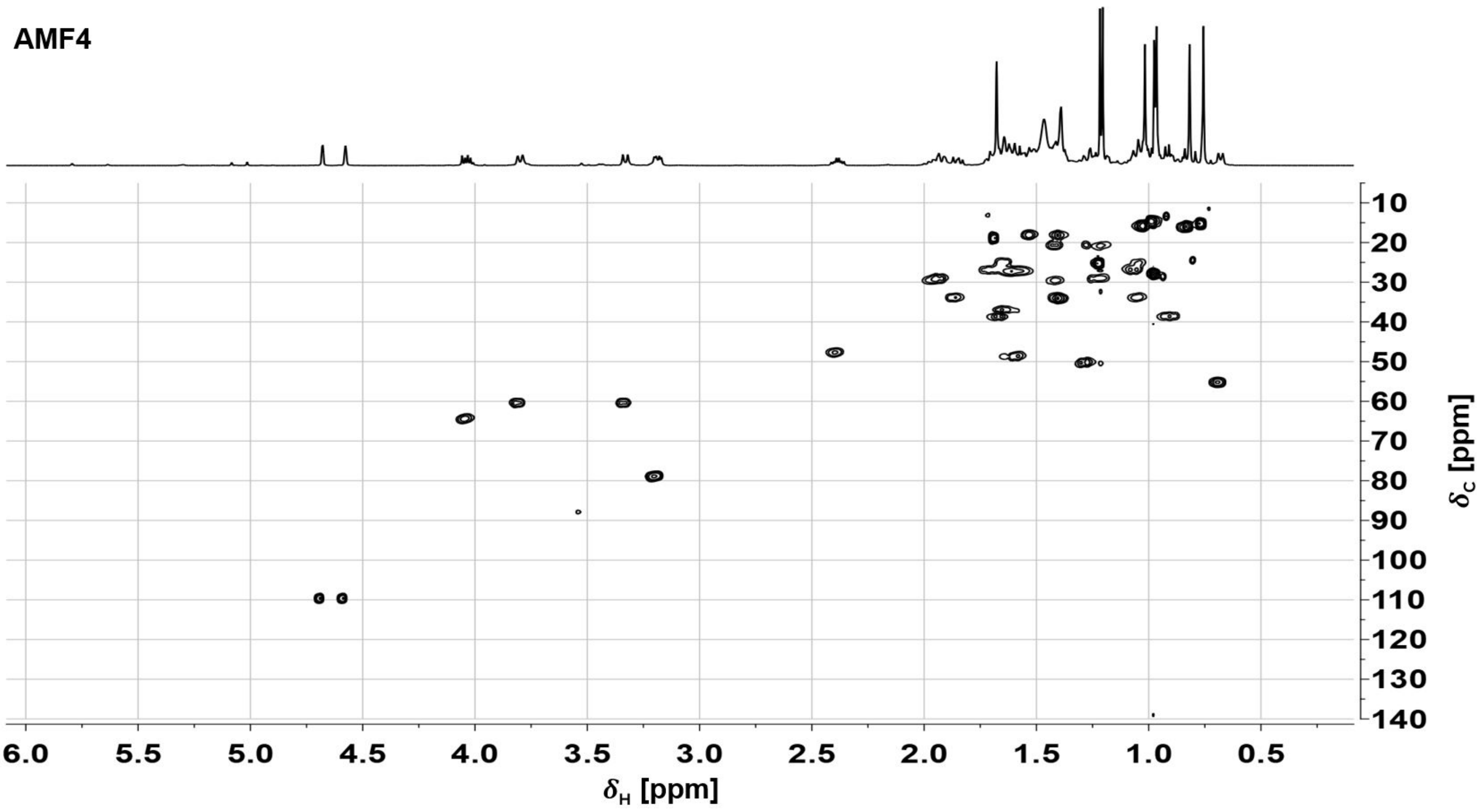

Figure S12. $^{1}H-^{13}C$ HSQC spectrum of AMF4.

The measurement was performed in deuterated chloroform-D1 at 500.19 MHz for ^{1}H NMR and 125.77 MHz for ^{13}C NMR.

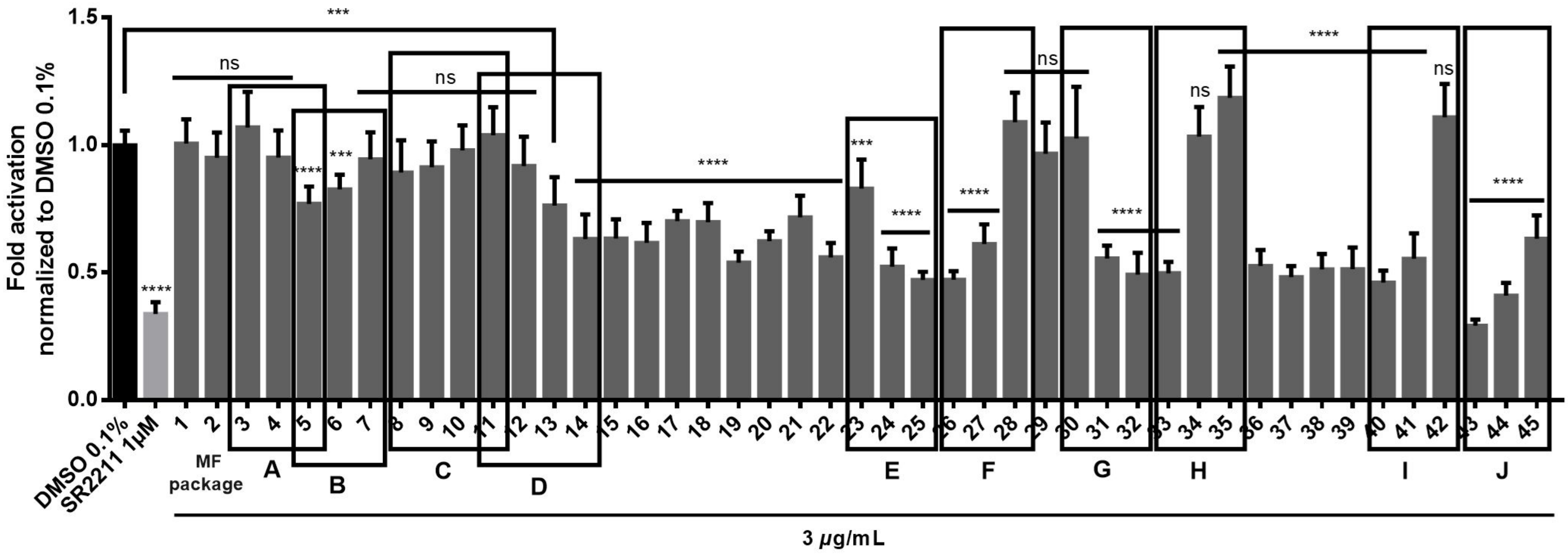

Figure S13. Inverse agonist activity of MF1–MF45 on RORγ.

The measurements were based on RORγ-Gal4 luciferase assays. The sample concentrations were 3 μg/mL. Results are expressed as fold activations, bars represent transactivation activities expressed as means ± standard deviation of three biological replicates (n = 3) measured in technical quadruplicates. One-way ANOVA followed by Dunnett's post hoc test were used for statistical analysis. **** $p \leq 0.0001$, *** $p \leq 0.001$, ns $p > 0.05$ compared to vehicle control. Packages A–J were selected based on their ascending or descending activity over a minimum of three consecutive MFs.

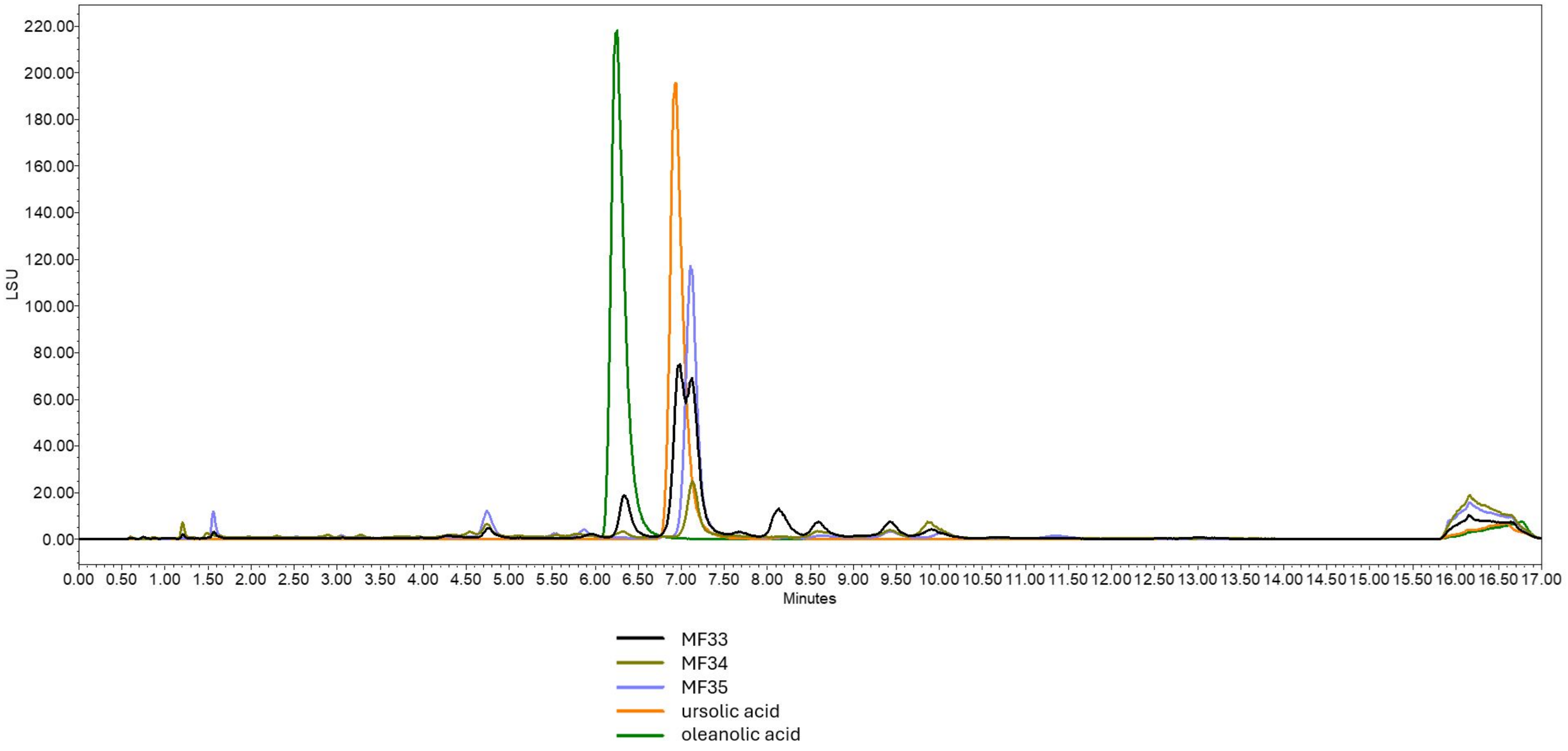

Figure S14. UHPSFC-ELSD results of MF33–35 and references **1** and **2**.

Ultra-high performance supercritical fluid chromatography (UHPSFC) results obtained via evaporative light scattering detector (ELSD) for microfractions MF33 (black), MF34 (olive), and MF35 (mauve) and reference compounds ursolic acid (**1**) (orange), oleanolic acid (**2**) (dark green).

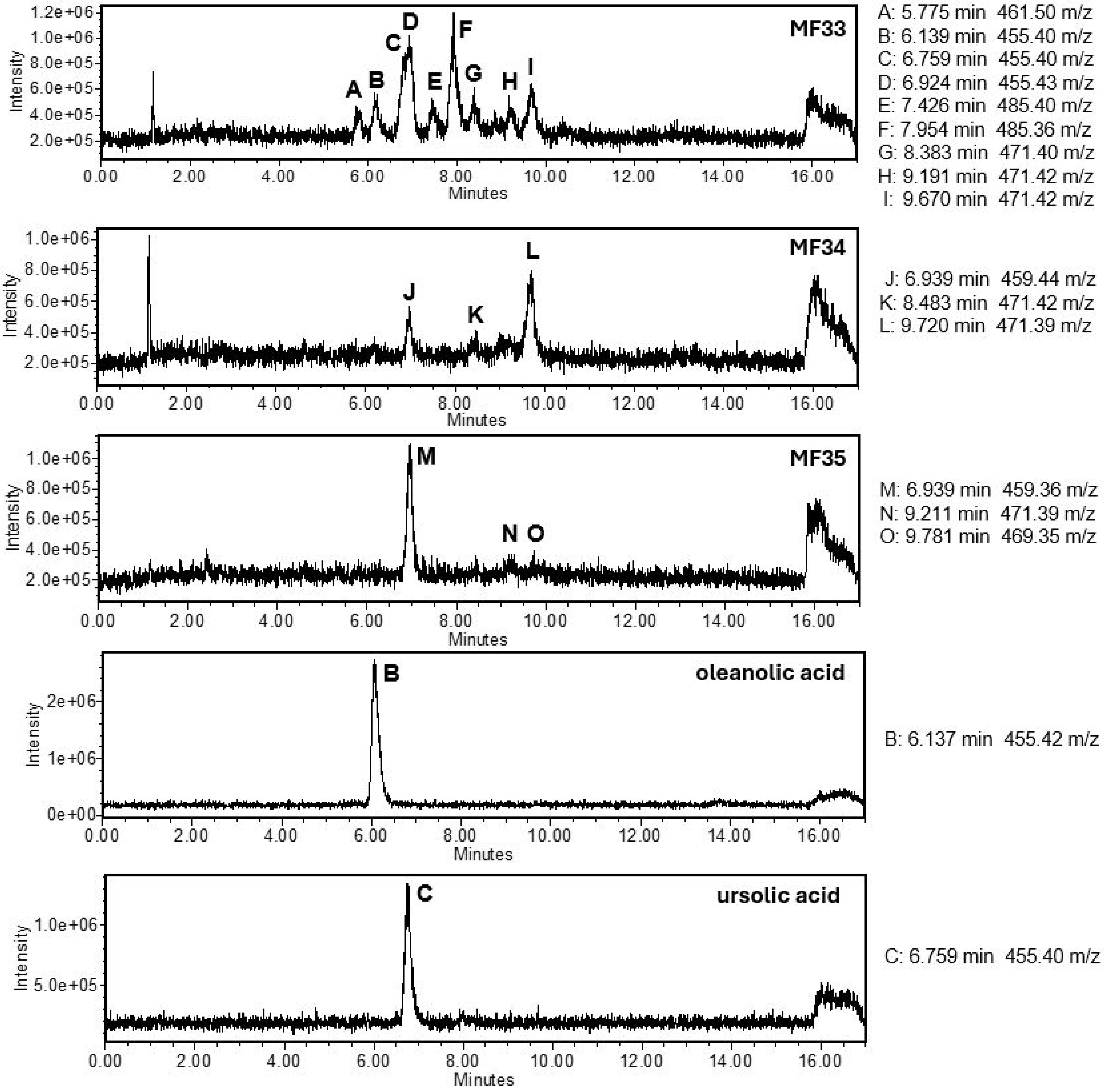

Figure S15. UHPSFC-MS results of MF33–35 and references **1** and **2**.

Ultra-high performance supercritical fluid chromatography (UHPSFC) results obtained via single quadrupole mass detector (Scan 100.00–1000.00 Da, negative ionization mode, cone voltage CV=30) for microfractions MF33, MF34, and MF35 and reference compounds ursolic acid (**1**), and oleanolic acid (**2**). Values for m/z are given for negative ionization mode.

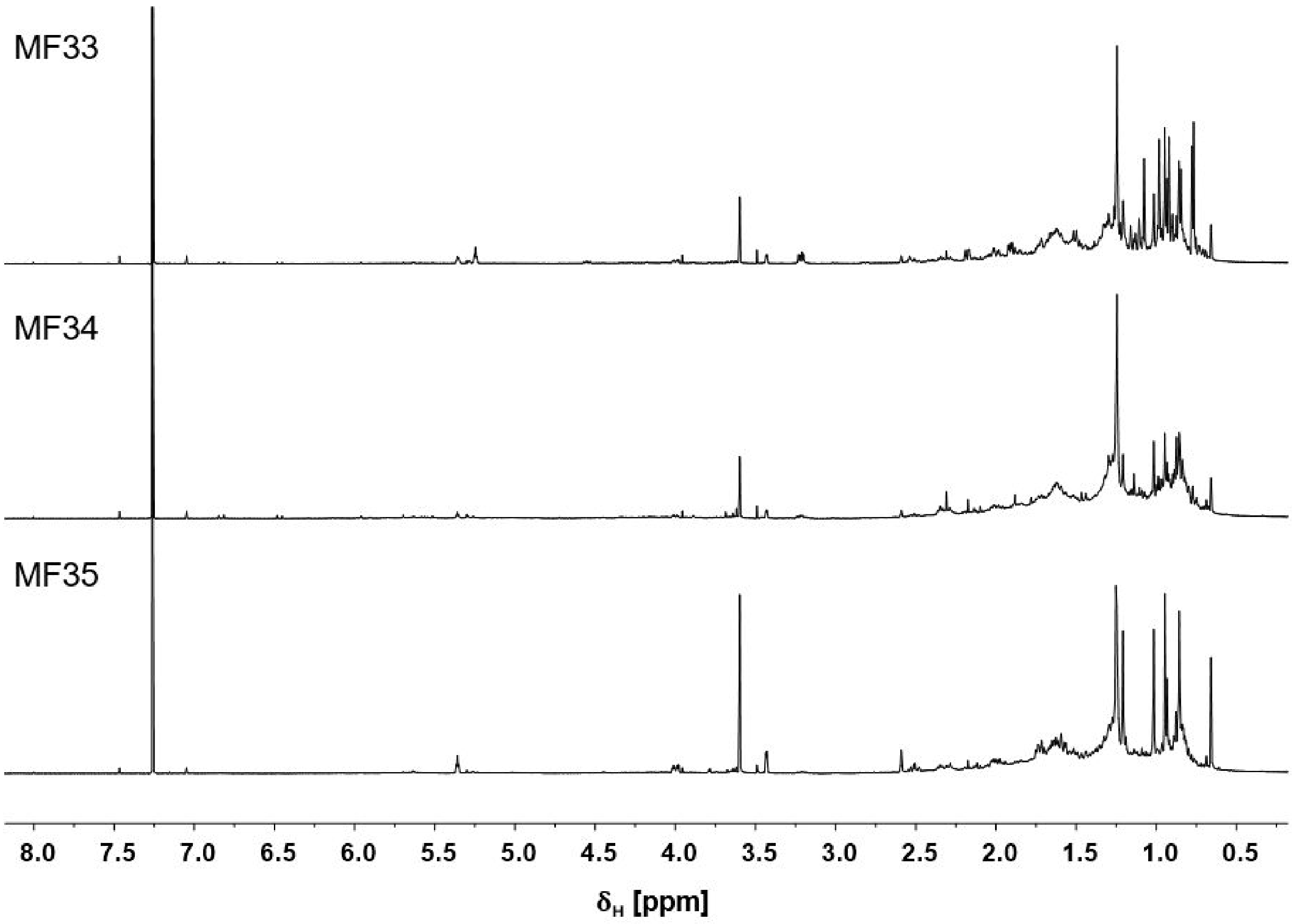

Figure S16. Stacked ^{1}H NMR plot of MF33–35.

The measurements were performed deuterated chloroform-D1 at 500.19 MHz.

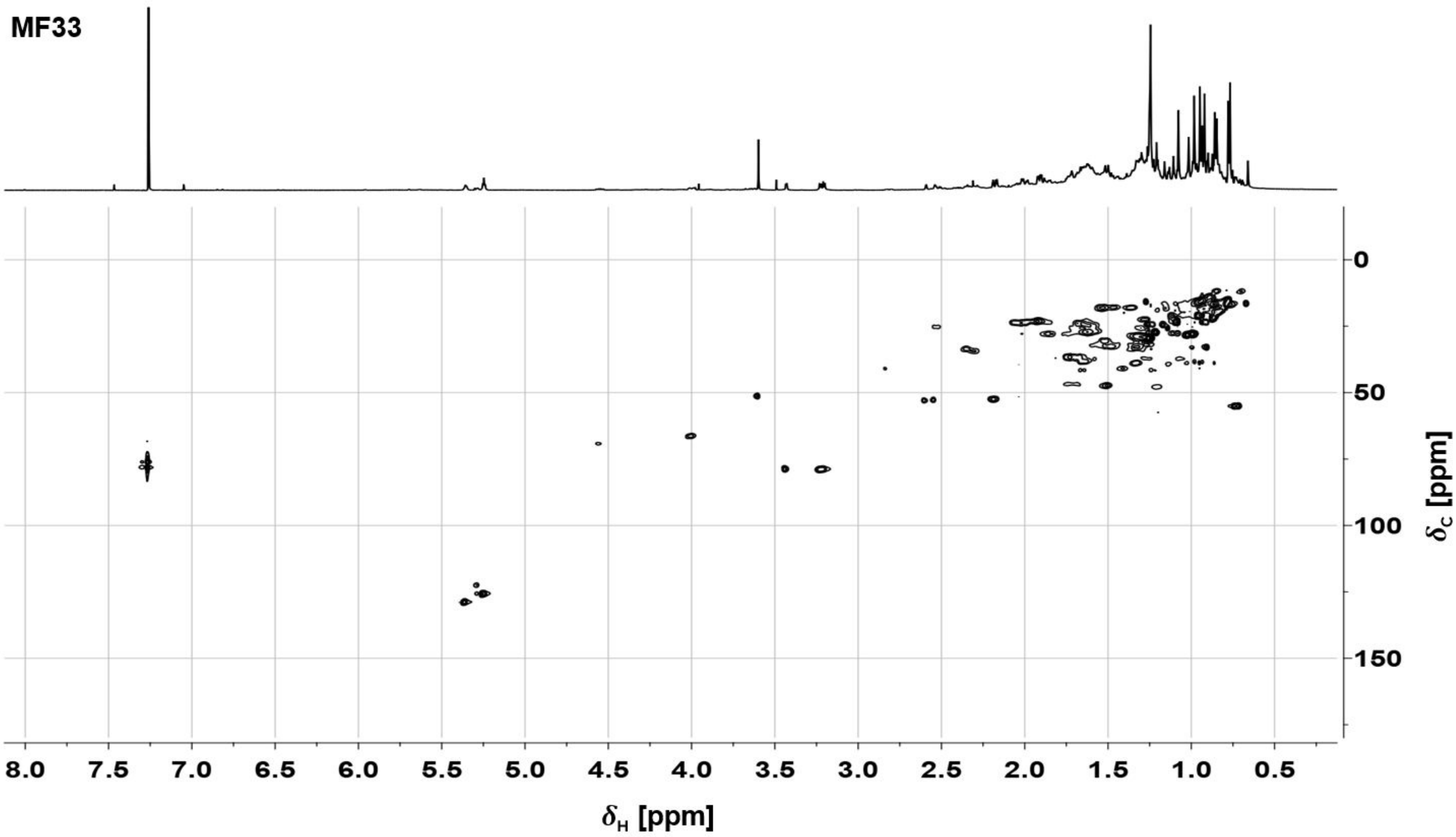

Figure S17. ^{1}H–^{13}C HSQC spectrum of MF33.

The measurement was performed in deuterated chloroform-D1 at 500.19 MHz for ^{1}H NMR and 125.77 MHz for ^{13}C NMR.

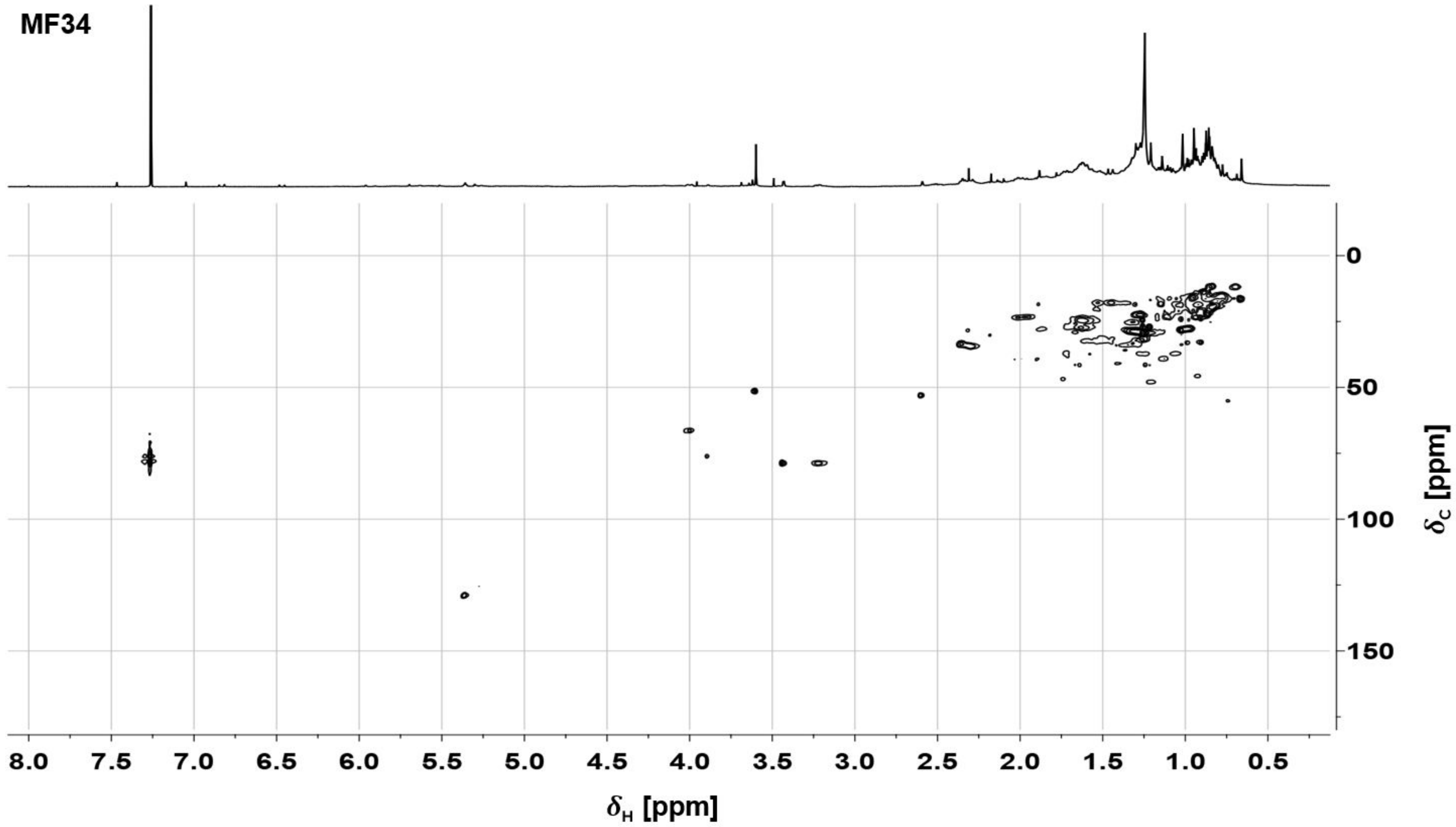

Figure S18. ^{1}H–^{13}C HSQC spectrum of MF34.

The measurement was performed in deuterated chloroform-D1 at 500.19 MHz for ^{1}H NMR and 125.77 MHz for ^{13}C NMR.

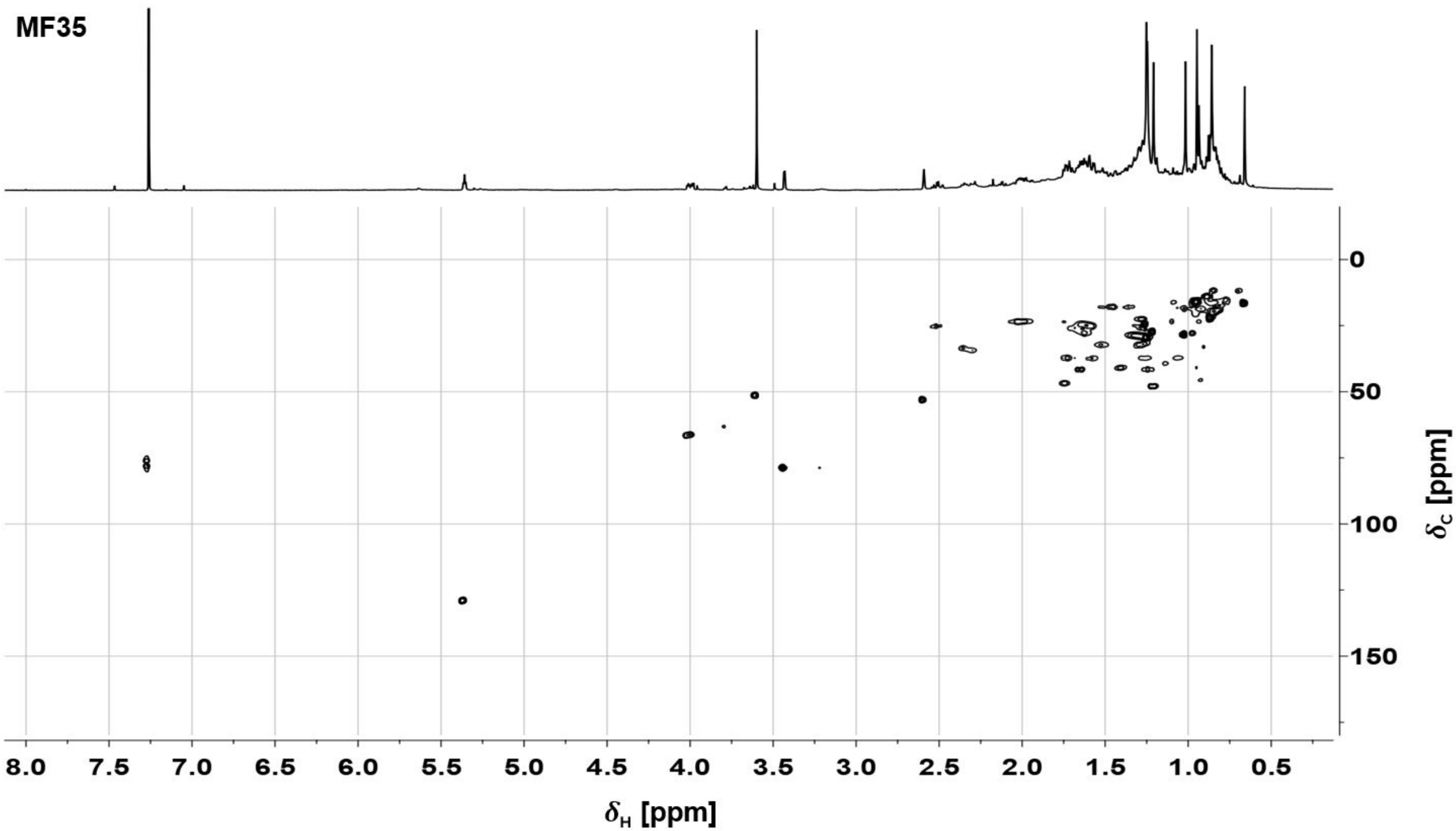

Figure S19. $^{1}H-^{13}C$ HSQC spectrum of MF35.

The measurement was performed in deuterated chloroform-D1 at 500.19 MHz for ^{1}H NMR and 125.77 MHz for ^{13}C NMR.

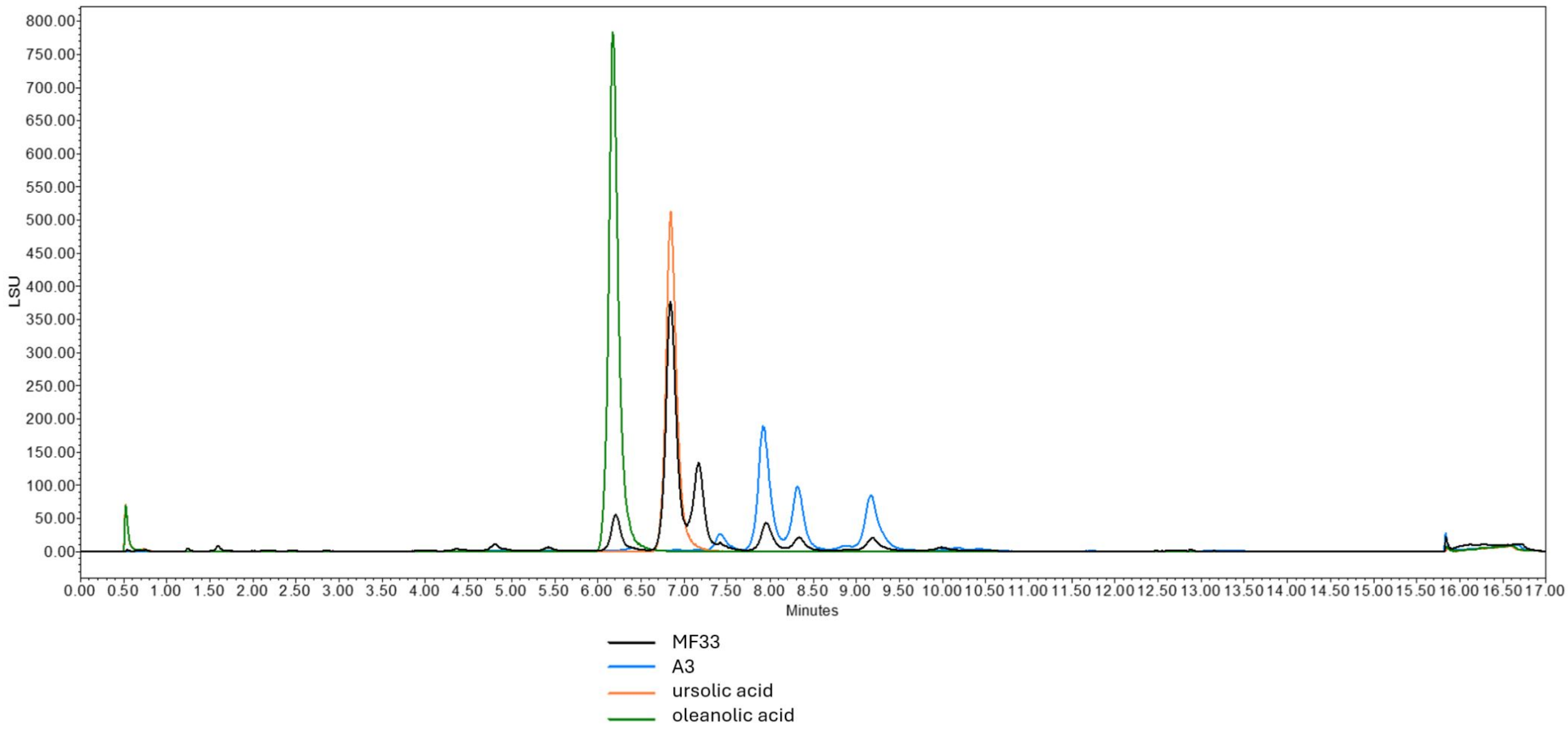

Figure S20. UHPSFC-ELSD chromatograms of MF33, A3, and compounds **1** and **2**.

Overlayed chromatograms of microfraction MF33, fraction A3, ursolic acid (**1**), and oleanolic acid (**2**) obtained via ultra-high performance supercritical fluid chromatography (UHPSFC), equipped with an evaporative light scattering detector (ELSD). While **1** (orange) and **2** (green) are present in MF33, after separation, fraction A3 does not contain both reference compounds.

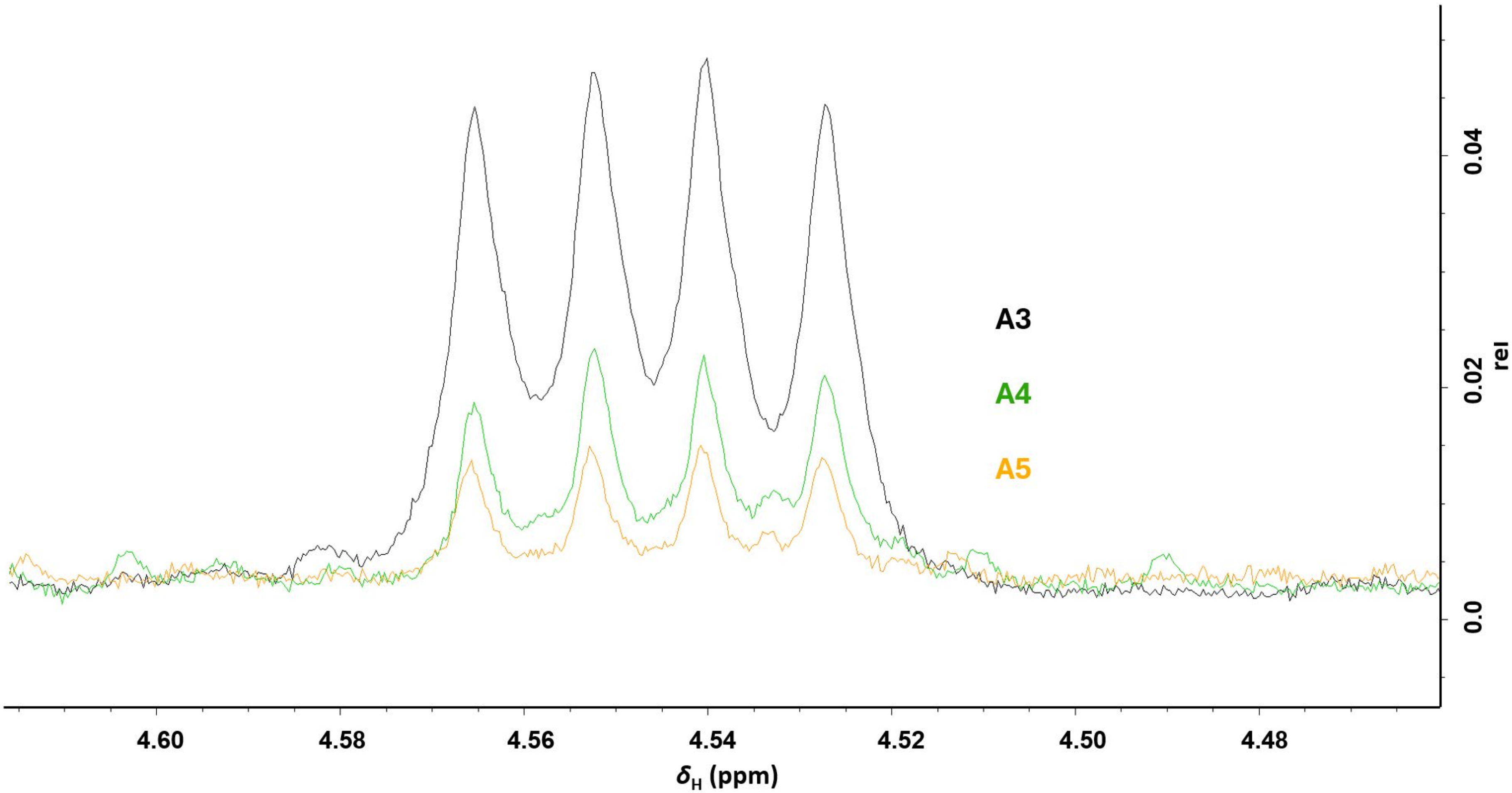

Figure S21. Overlayed ^{1}H NMR of A3–A5 at δ_H 4.55.

Proton NMR signal of the marker feature at δ_H 4.55, measured in deuterated chloroform-D1 at 500.19 MHz, with decreasing intensity from fraction A3 (black), to fraction A4 (green), to fraction A5 (orange).

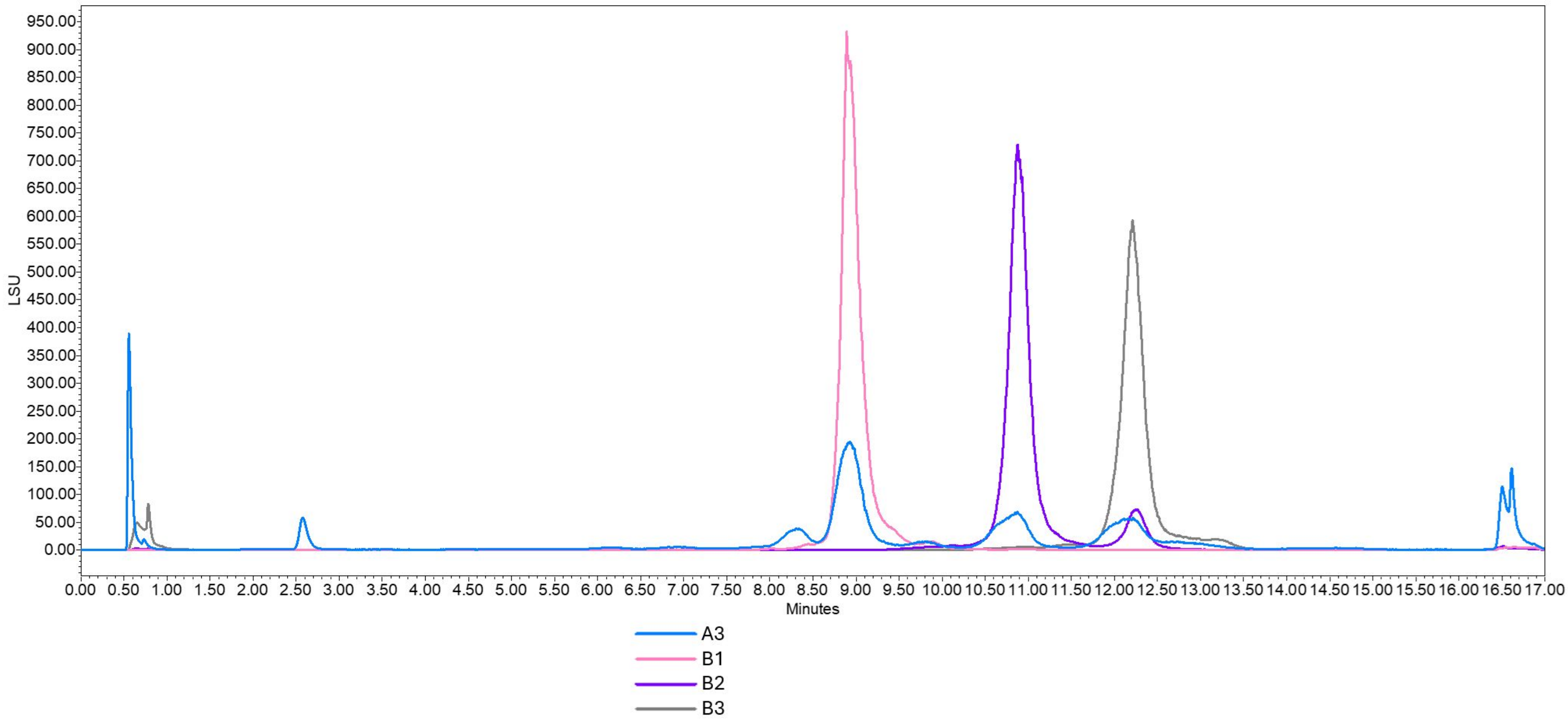

Figure S22. UHPSFC-ELSD results for A3, and B1–B3.

Ultra-high performance supercritical fluid chromatography (UHPSFC) results of an evaporative light scattering detector (ELSD) for fraction A3 (light blue) and its sub-fractions B1 (old rose), B2 (violet), and B3 (gray), obtained via semi-preparative SFC.

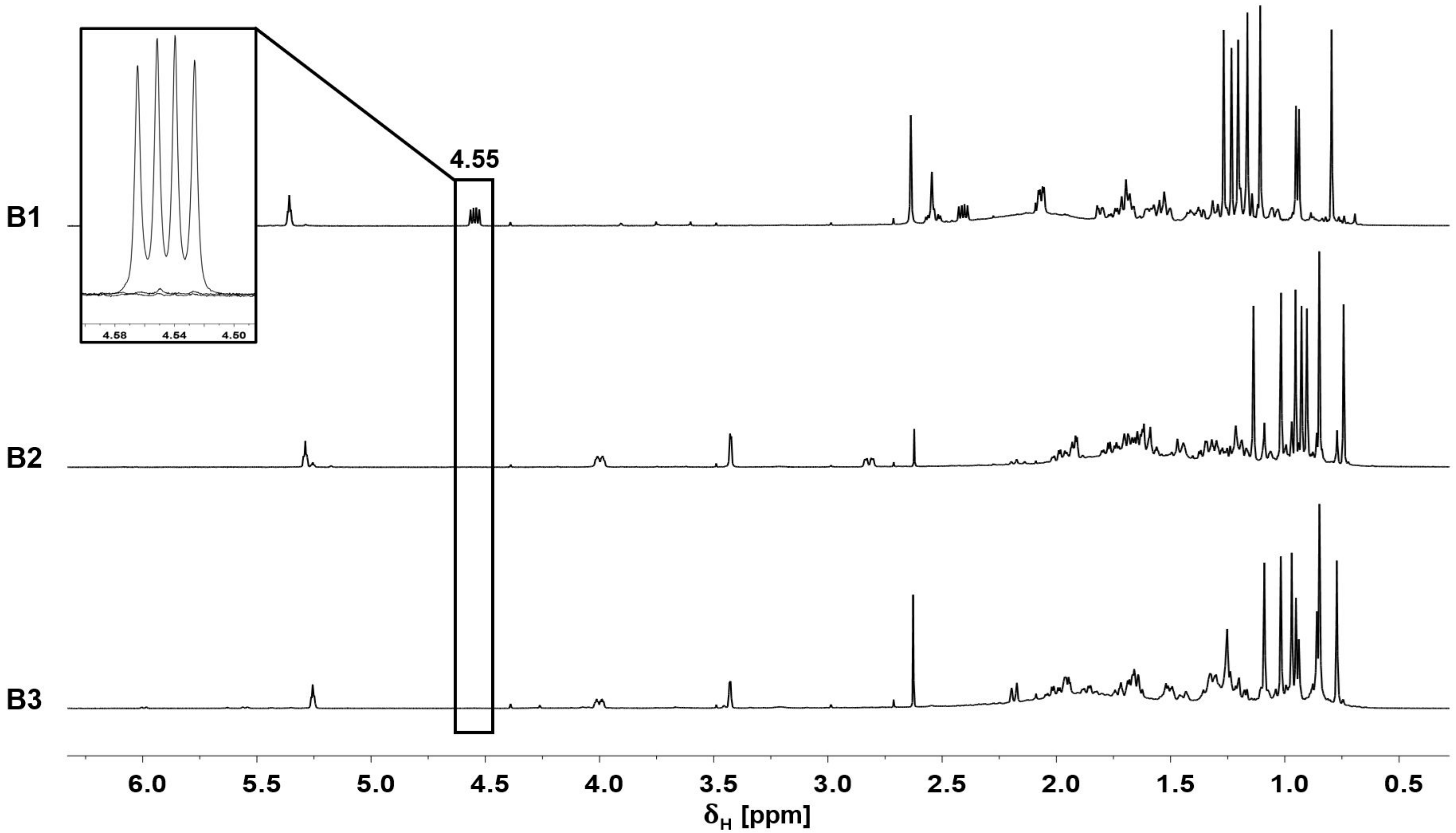

Figure S23. Stacked plot of the ^{1}H NMR spectra of fractions B1–B3.

The measurement was performed in deuterated chloroform-D1 at 500.19 MHz. The area of the doublet of doublets marker signal in the three spectra is marked with a black rectangle, with a zoom into the superimposed spectra at the respective δ_H region at δ_H 4.55.

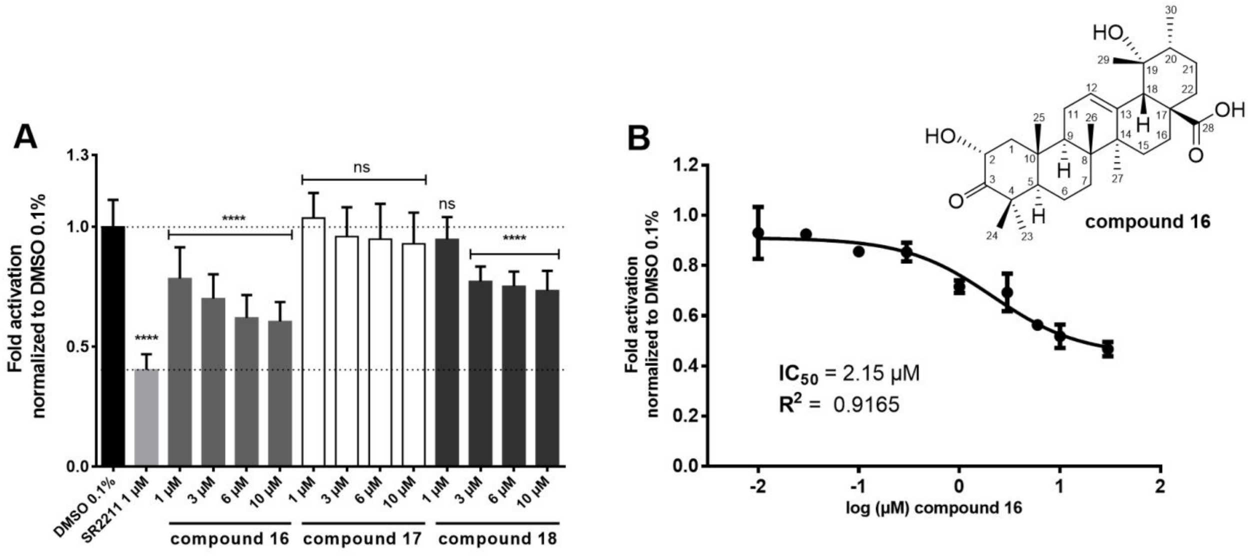

Figure S24. Bioactivity testing of compounds **16**, **17**, and **18** on RORγ. (A) RORγ inverse agonistic activities of 2α,19α-dihydroxy-3-oxo-12-ursen-28-oic acid (**16**), 3-epimaslinic acid (**17**), and 3-epicorosolic acid (**18**) in RORγ-Gal4 luciferase reporter gene assays. Bars (A) and data points (B) represent the mean ± standard deviation of three biological replicates (n = 3), measured in technical quadruplicates. One-way ANOVA followed by Dunnett's post hoc test were used for statistical analysis. **** $p \leq$ 0.0001, *** $p \leq$ 0.001, ns $p >$ 0.05 compared to vehicle control. (B) Concentration-response curve for the RORγ inverse agonistic activity of compound **16**. The IC_{50} value of **16** was determined using nonlinear regression.

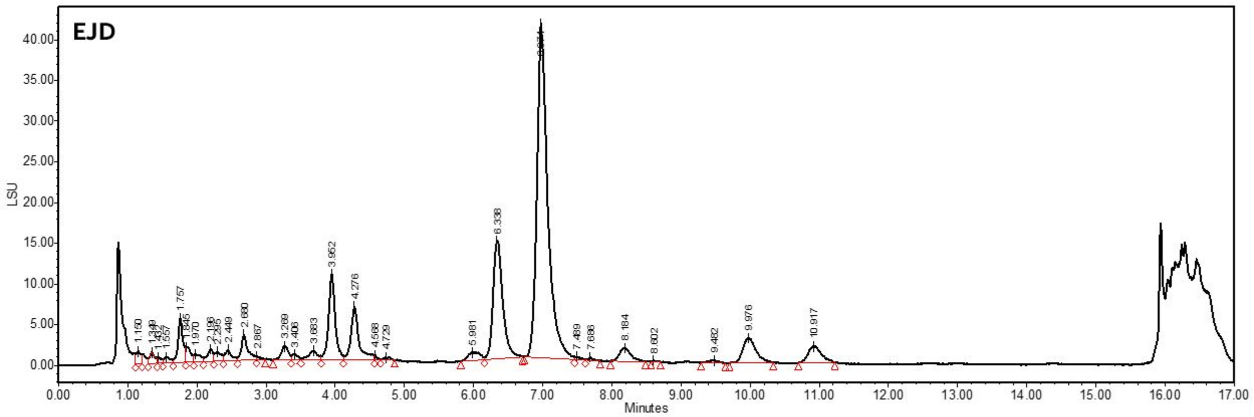

retention time [min]	area [µV*sec]	area [%]		retention time [min]	area [µV*sec]	area [%]
1.150	7703	0.80		3.952	70303	7.33
1.349	7093	0.74		4.276	53151	5.54
1.432	2763	0.29		4.568	1665	0.17
1.557	4628	0.48		4.729	2234	0.23
1.757	26841	2.80		5.981	12646	1.32
1.845	9365	0.98		6.338	141280	14.73
1.970	6544	0.68		6.974	443721	46.28
2.196	8209	0.86		7.489	2175	0.23
2.295	7734	0.81		7.686	1670	0.17
2.449	8905	0.93		8.184	18970	1.98
2.680	20177	2.10		8.602	467	0.05
2.867	1548	0.16		9.482	3768	0.39
3.269	12613	1.32		9.976	40839	4.26
3.406	4153	0.43		10.917	26630	2.78
3.683	11061	1.15				

Figure S25. UHPSFC-ELSD results for EJD with integrated area under the curves.

Ultra-high performance supercritical fluid chromatography (UHPSFC) results of an evaporative light scattering detector (ELSD) for *Eriobotrya japonica* dichloromethane crude extract (EJD), with integrated area under the curves. Proportions of the signals are given in the table included. EJD contained 1.98 % of compound **16** (at min 8.184), 0.05 % of compound **17** (at min 8.602), and 0.39 % of compound **18** (at min 9.482).

Supporting Information References

(1) Ladurner, A.; Schwarz, P. F.; Dirsch, V. M. Natural products as modulators of retinoic acid receptor-related orphan receptors (RORs). *Nat Prod Rep* **2021**, *38* (4), 757-781. DOI: 10.1039/d0np00047g

(2) Pastwinska, J.; Karas, K.; Salkowska, A.; Karwaciak, I.; Chalaskiewicz, K.; Wojtczak, B. A.; Bachorz, R. A.; Ratajewski, M. Identification of Corosolic and Oleanolic Acids as Molecules Antagonizing the Human RORgammaT Nuclear Receptor Using the Calculated Fingerprints of the Molecular Similarity. *Int J Mol Sci* **2022**, *23* (3), 1906. DOI: 10.3390/ijms23031906

(3) Guan, D.; Li, Y.; Zhao, X.; Wang, K.; Guo, Y.; Dong, N.; Cui, Y.; Gao, Y.; Wang, M.; Wang, J.; Ren, Y.; Shang, P.; Liu, Y. Hederagenol improves multiple sclerosis by modulating Th17 cell differentiation. *IUBMB Life* **2024**, *76* (10), 845-857. DOI: 10.1002/iub.2863

(4) Genet, C.; Strehle, A.; Schmidt, C.; Boudjelal, G.; Lobstein, A.; Schoonjans, K.; Souchet, M.; Auwerx, J.; Saladin, R.; Wagner, A. Structure-activity relationship study of betulinic acid, a novel and selective TGR5 agonist, and its synthetic derivatives: potential impact in diabetes. *J Med Chem* **2010**, *53* (1), 178-190. DOI: 10.1021/jm900872z

(5) Lo, S. H.; Li, Y.; Cheng, K. C.; Niu, C. S.; Cheng, J. T.; Niu, H. S. Ursolic acid activates the TGR5 receptor to enhance GLP-1 secretion in type 1-like diabetic rats. *Naunyn Schmiedebergs Arch Pharmacol* **2017**, *390* (11), 1097-1104. DOI: 10.1007/s00210-017-1409-9

(6) Sato, H.; Genet, C.; Strehle, A.; Thomas, C.; Lobstein, A.; Wagner, A.; Mioskowski, C.; Auwerx, J.; Saladin, R. Anti-hyperglycemic activity of a TGR5 agonist isolated from Olea europaea. *Biochem Biophys Res Commun* **2007**, *362* (4), 793-798. DOI: 10.1016/j.bbrc.2007.06.130

(7) Ladurner, A.; Zehl, M.; Grienke, U.; Hofstadler, C.; Faur, N.; Pereira, F. C.; Berry, D.; Dirsch, V. M.; Rollinger, J. M. Allspice and Clove As Source of Triterpene Acids Activating the G Protein-Coupled Bile Acid Receptor TGR5. *Front Pharmacol* **2017**, *8*, 468. DOI: 10.3389/fphar.2017.00468

(8) Kirchweger, B.; Kratz, J. M.; Ladurner, A.; Grienke, U.; Langer, T.; Dirsch, V. M.; Rollinger, J. M. In Silico Workflow for the Discovery of Natural Products Activating the G Protein-Coupled Bile Acid Receptor 1. *Front Chem* **2018**, *6*, 242. DOI: 10.3389/fchem.2018.00242

(9) Xu, T.; Wang, X.; Zhong, B.; Nurieva, R. I.; Ding, S.; Dong, C. Ursolic acid suppresses interleukin-17 (IL-17) production by selectively antagonizing the function of RORgamma t protein. *J Biol Chem* **2011**, *286* (26), 22707-22710. DOI: 10.1074/jbc.C111.250407

(10) Pastwinska, J.; Karas, K.; Salkowska, A.; Karwaciak, I.; Chalaskiewicz, K.; Wojtczak, B. A.; Bachorz, R. A.; Ratajewski, M. Identification of Corosolic and Oleanolic Acids as Molecules Antagonizing the Human RORgammaT Nuclear Receptor Using the Calculated Fingerprints of the Molecular Similarity. *Int J Mol Sci* **2022**, *23* (3). DOI: 10.3390/ijms23031906

(11) Schwarz, P. F.; Perhal, A. F.; Preglej, T.; Breit, L.; Schmetterer, K. G.; Grienke, U.; Janneschütz, J.; Chen, Y.; Rochel, N.; Kirchmair, J.; Schützenmeister, N.; Rollinger, J. M.; Bonelli, M.; Dirsch, V. M. Primulagenin A is a potent inverse agonist of the nuclear receptor RAR-related orphan receptor gamma (RORγ). *bioRxiv 2025.04.01.646598* **2025**. DOI: 10.1101/2025.04.01.646598

(12) Zhou, X.; Chen, H.; Wei, F.; Zhao, Q.; Su, Q.; Lei, Y.; Yin, M.; Tian, X.; Liu, Z.; Yu, B.; Bai, C.; He, X.; Huang, Z. The Inhibitory Effects of Pentacyclic Triterpenes from Loquat Leaf against Th17 Differentiation. *Immunol Invest* **2020**, *49* (6), 632-647. DOI: 10.1080/08820139.2019.1698599

(13) Guan, D.; Li, Y.; Zhao, X.; Wang, K.; Guo, Y.; Dong, N.; Cui, Y.; Gao, Y.; Wang, M.; Wang, J.; Ren, Y.; Shang, P.; Liu, Y. Hederagenol improves multiple sclerosis by modulating Th17 cell differentiation. *IUBMB Life* **2024**. DOI: 10.1002/iub.2863
